# Identification of ORC1/CDC6-Interacting Factors in *Trypanosoma brucei* Reveals Critical Features of Origin Recognition Complex Architecture

**DOI:** 10.1371/journal.pone.0032674

**Published:** 2012-03-08

**Authors:** Calvin Tiengwe, Lucio Marcello, Helen Farr, Catarina Gadelha, Richard Burchmore, J. David Barry, Stephen D. Bell, Richard McCulloch

**Affiliations:** 1 The Wellcome Trust Centre for Molecular Parasitology, Institute of Infection, Immunity and Inflammation, University of Glasgow, Glasgow, United Kingdom; 2 Sir William Dunn School of Pathology, University of Oxford, Oxford, United Kingdom; 3 Sir Henry Wellcome Functional Genomics Facility, Institute of Infection, Immunity and Inflammation, University of Glasgow, Glasgow, United Kingdom; The University of Nottingham, United Kingdom

## Abstract

DNA Replication initiates by formation of a pre-replication complex on sequences termed origins. In eukaryotes, the pre-replication complex is composed of the Origin Recognition Complex (ORC), Cdc6 and the MCM replicative helicase in conjunction with Cdt1. Eukaryotic ORC is considered to be composed of six subunits, named Orc1–6, and monomeric Cdc6 is closely related in sequence to Orc1. However, ORC has been little explored in protists, and only a single ORC protein, related to both Orc1 and Cdc6, has been shown to act in DNA replication in *Trypanosoma brucei*. Here we identify three highly diverged putative *T. brucei* ORC components that interact with ORC1/CDC6 and contribute to cell division. Two of these factors are so diverged that we cannot determine if they are eukaryotic ORC subunit orthologues, or are parasite-specific replication factors. The other we show to be a highly diverged Orc4 orthologue, demonstrating that this is one of the most widely conserved ORC subunits in protists and revealing it to be a key element of eukaryotic ORC architecture. Additionally, we have examined interactions amongst the *T. brucei* MCM subunits and show that this has the conventional eukaryotic heterohexameric structure, suggesting that divergence in the *T. brucei* replication machinery is limited to the earliest steps in origin licensing.

## Introduction

Genome replication is central to the propagation of all life. In DNA genomes, replication initiates by the designation of genome sequences as origins, where synthesis of a copy of the genetic material begins. Origin designation is a complex, tightly regulated process whose core mechanisms and machinery are conserved between eukaryotes and archaea [1], [2]. This reaction involves the formation of a pre-replication complex (pre-RC), which in eukaryotes comprises the Origin Recognition Complex (ORC), Cdc6, Cdt1 and the replicative MCM helicase; for reviews, see [3]–[6]. ORC is frequently described as being composed of six subunits, named Orc1–6, in all eukaryotes that have been examined to date [7]. It is the earliest acting pre-RC component during DNA replication origin designation, being responsible for binding to origins. ORC was first purified from *Saccharomyces cerevisiae* [8], and the subunits were numbered in descending order of size. Orthologues of each subunit have subsequently been identified from a range of eukaryotes [7]. Orcs1–5 each display sequence conservation with the AAA^+^ family of ATPases, including Walker A and B box nucleotide binding domains and sensor motifs involved in nucleotide hydrolysis [9]. Winged helix domains are normally found at the C-termini of these subunits, which may mediate DNA binding in association with the AAA+ ATPase domain [10]–[12]. Orc1 in most eukaryotes also possesses N-terminal homology with Sir3, which is involved in transcriptional silencing, including a bromo-adjacent homology (BAH) domain [13]. Orc6 is more poorly conserved amongst eukaryotes [14] and appears unrelated to the other ORC subunits, lacking discernible homology with AAA+ domains [7]. ATP binding and hydrolysis by the ORC subunits causes conformational changes associated with ORC assembly and DNA binding, as well as modulating interaction with the other pre-RC components [12], [15]–[18].

Cdc6 (Cell Division Cycle 6) was first identified in *S. cerevisiae* [19], where the gene is transcribed only in late G_1_ and early S-phase [20]. It is an AAA+ ATPase closely related to Orc1, also possessing a C-terminal winged helix domain, and phylogenetics suggest the proteins arose from a common ancestor [7], [21]. In yeast, Cdc6 is recruited to ORC bound to origin DNA, and the protein's ATPase activity modulates the structure of the ternary complex [12], [16], [22]–[24]. A consequence of this binding is the recruitment of Cdt1 (Cdc10-dependent transcript 1), a protein originally identified in *Schizosaccharomyces pombe* whose expression is cell cycle-regulated [25]. Cdt1 has been identified in a number of eukaryotes, but the sequences show only low levels of sequence homology and lack clear enzymatic motifs [26]. Cdt1 acts as an adaptor protein that mediates interaction between the MCM helicase and the ORC-Cdc6 complex [27], [28] and, at least in yeast, forms a stable complex with MCM [24]. In eukaryotes, the replicative MCM (Mini Chromosome Maintenance) helicase is composed of six conserved subunits, named Mcm2–7, which form a hetero-hexameric ring on DNA. Binding of MCM completes the pre-RC complex and ‘licenses’ origins to be replicated. Subsequent steps involve the binding of further factors, including DNA polymerases [4].

The pre-RC machinery of archaea is evolutionarily conserved with that of eukaryotes, though appears to be simpler in architecture [1], [2]. A single protein, Orc1/Cdc6, fulfils the functions of eukaryotic ORC and Cdc6. In some archaeal species only a single Orc1/Ccd6 gene is found, while in others greater numbers are present [29]. Characterised archaeal Orc1/Cdc6 proteins bind in a sequence-specific manner to replication origins, possess ATPase activity that may be due to co-operative interactions between monomers, and distort the origin DNA on binding, suggesting they designate origins in similar ways to eukaryotic ORC [10], [11]. The archaeal MCM helicase is a homohexamer, and thus structurally distinct from the eukaryotic heterohexamer [30]. An orthologue of Cdt1 has not been described in archaea, though a protein named Winged helix initiator Protein (WhiP) has been identified that possesses sequence similarity with Cdt1 and has been shown to bind origins [31]. Whether this acts analogously to eukaryotic Cdt1 is currently unclear, and direct interaction between Orc1/Cdc6 and MCM has been described in a number of archaeal species, perhaps suggesting that recruitment in these organisms may not need a Cdt1-like adaptor [32]–[34].

In protists, unicellular microbes that represent much of the diversity of the eukaryotic kingdom and include a number of important pathogens [35], [36], the pre-RC machinery has been examined only to a limited extent. In the apicomplexan parasite *Plasmodium falciparum*, functional analysis has examined two components of a putative ORC complex, Orc1 and Orc5 [37], [38]. *P. falciparum* Orc1 appears to be significantly enlarged relative to most eukaryotic Orc1 orthologues, possessing N- and C-terminal extensions, and co-localises with MCM during replicative life cycle stages [39]. In *Tetrahymena thermophila* a multisubunit ORC complex has also been described [40], [41]. In contrast, bioinformatic analyses of the genomes of *T. brucei* and related kinetoplastid parasites identified only a single ORC-related protein [21], [42], [43]. This protein contains well-conserved AAA+ ATPase motifs and is related in sequence to both Orc1 and Cdc6, though lacks N-terminal sequences found in other eukaryotic Orc1 subunits, including the BAH domain. The structural similarity of the kinetoplastid proteins to Orc1/Cdc6 in archaea has led to their re-naming as ORC1/CDC6 [43], [44]. TbORC1/CDC6 is able to complement *S. cerevisiae cdc6* temperature sensitive mutants (but not *orc1* mutants), arguing the protein has Cdc6-related properties [43]. Given this finding, and the apparent absence of further ORC subunits, the possibility that origin designation in these parasites may be archaeal-like has been discussed [21]. However, a factor, named ORC1b, was identified very recently that contains conserved ATPase motifs and interacts with ORC1/CDC6, perhaps suggesting a larger ORC [45]. Here, we describe the identification of three further *T. brucei* proteins that interact with TbORC1/CDC6. One of these factors we show to be a diverged orthologue of Orc4, suggesting that this is a core, conserved component of eukaryotic ORC. This finding suggests that an ORC is present in *T. brucei*, though it is highly diverged. In support of this, we show that RNAi knockdown TbORC1/CDC6 or any of the three interacting factors results in striking phenocopying in both tsetse fly- and mammal-infective *T. brucei*, suggesting functional overlap. Finally, we describe a sub-complex of the *T. brucei* MCM helicase, which suggests a conserved topology with other eukaryotic MCM helicases.

## Results

### The *T. brucei* MCM helicase is a conserved heterohexamer

In eukaryotes Cdc6 and Cdt1 function to recruit the MCM helicase complex for local DNA unwinding by mediating interaction with ORC [6]. Homology searches have failed to identify a Cdt1 homologue in any trypanosomatid genome [21], [42]. A mechanistic consequence of such a putative absence could be that MCM in *T. brucei* is recruited directly by TbORC1/CDC6. If correct, this would lend support to the suggestion that the very earliest steps in origin designation in *T. brucei* may be archaeal-like, and may relate to the potential that TbORC1/CDC6 provides both Cdc6 and Orc1 functions. Such direct interaction between one or more subunits of ORC and MCM has not been reported in eukaryotes [28], but has been seen between archaeal ORC1/CDC6 and the replicative helicase in a number of species by different methods [32]–[34]. We therefore decided to test this experimentally in *T. brucei*.

**Figure 1:**
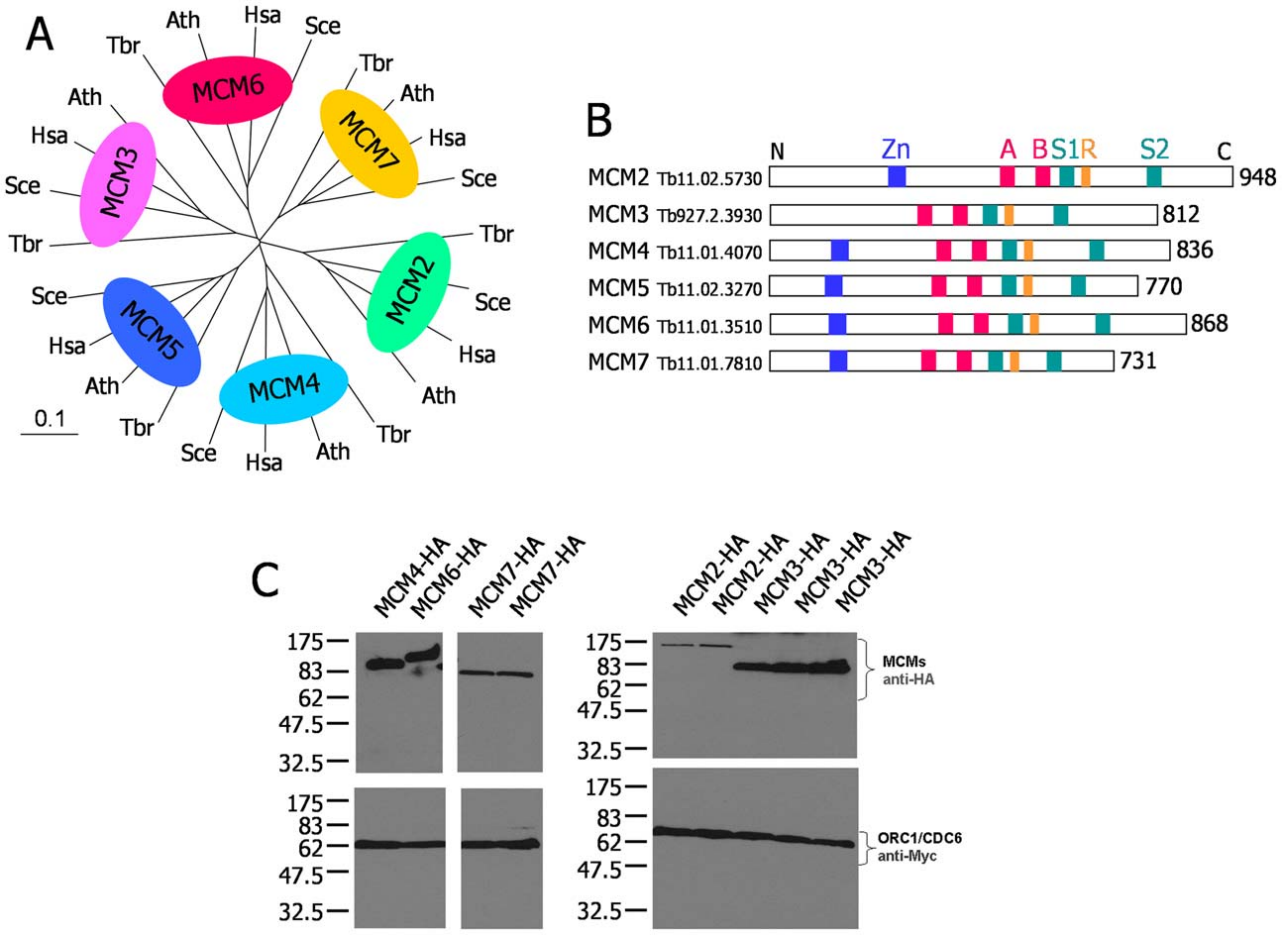
MCM helicase subunits in *T. brucei* and co-expression with ORC1/CDC6 as epitope tagged variants. **A.** An unrooted phylogenetic tree is shown, detailing the homology between predicted MCM helicase subunits in *T. brucei* (Tbr) relative to orthologues in *H. sapiens* (Hsa), *S. cerevisiae* (Sce) and *A. thaliana* (Ath). Complete protein sequences were aligned with ClustalX, using default settings, and the phylogenetic tree was displayed using TreeView (Page, 1996); the distance corresponding to 10 amino acid changes per 100 positions is indicated (0.1) **B.** A diagrammatic representation of the MCM helicase subunits in *T. brucei*. The length of the predicted polypeptides is shown (in amino acid residues), and the position of conserved functional motifs are indicated: an N-terminal Zinc Finger (Zn, blue box); and Walker A and B boxes (A and B, red boxes), an Arginine finger (R, orange box) and sensor 1 and 2 motifs (S1 and S2, green boxes), all involved in nucleotide binding and hydrolysis. **C.** Western blots of procyclic form TREU 927 *T. brucei* cells co-expressing C-terminally HA-tagged TbMCM subunits (MCM-HA) and C-terminally Myc-tagged TbORC1/CDC6. The upper panel shows TbMCM-HA expression in whole cell extracts, detected using anti-HA antibody, and the bottom panel shows TbORC1/CDC6-Myc from the same whole cell extracts detected using anti-Myc antibody. Single clones are shown for TbMCM4-HA and TbMCM6-HA, two clones for TbMCM2-HA and TbMCM7-HA, and three clones for TbMCM3-HA. Size markers (kDa) are indicated.

Unlike in archaea, where the replicative helicase is a homohexamer [2], orthologues of all six core eukaryotic MCM subunits, named Mcm2–7 [4], can be unambiguously identified in the *T. brucei* genome (Fig. 1A,B). In addition, putative MCM8 and MCM9 orthologues appear to be present [46]. To test if TbORC1/CDC6 directly interacts with the MCM2–7 helicase, we generated constructs that allow us to C-terminally tag TbORC1/CDC6 with 12 copies of the Myc epitope, and to C-terminally tag each of the MCM subunits with six copies of the HA epitope. We first generated procyclic form (PCF) cells expressing TbORC1/CDC6-Myc, and these were then transformed individually with the six MCM subunit tagging constructs. Clones were obtained that expressed proteins reacting with anti-HA antiserum that were of the size expected for HA-tagged variants of each of the TbMCM subunits (Fig. 1C). In each case, co-expression of TbORC1/CDC6-Myc was confirmed by hybridisation with anti-Myc antibody (Fig. 1C). From the western blots it is evident that the individual HA-tagged TbMCM subunits were detected at different levels by the anti-HA antisera (with TbORC1/CDC6-Myc serving as a loading control). Visual inspection indicated TbMCM2 generated the weakest signal, followed by TbMCM7, and TbMCM3, -4 and -6 gave the same, higher levels. Clones expressing HA-MCM5 were recovered later and the level of expression of this protein appeared low relative to the other MCM subunits (data not shown); these clones were not analysed further.

**Figure 2:**
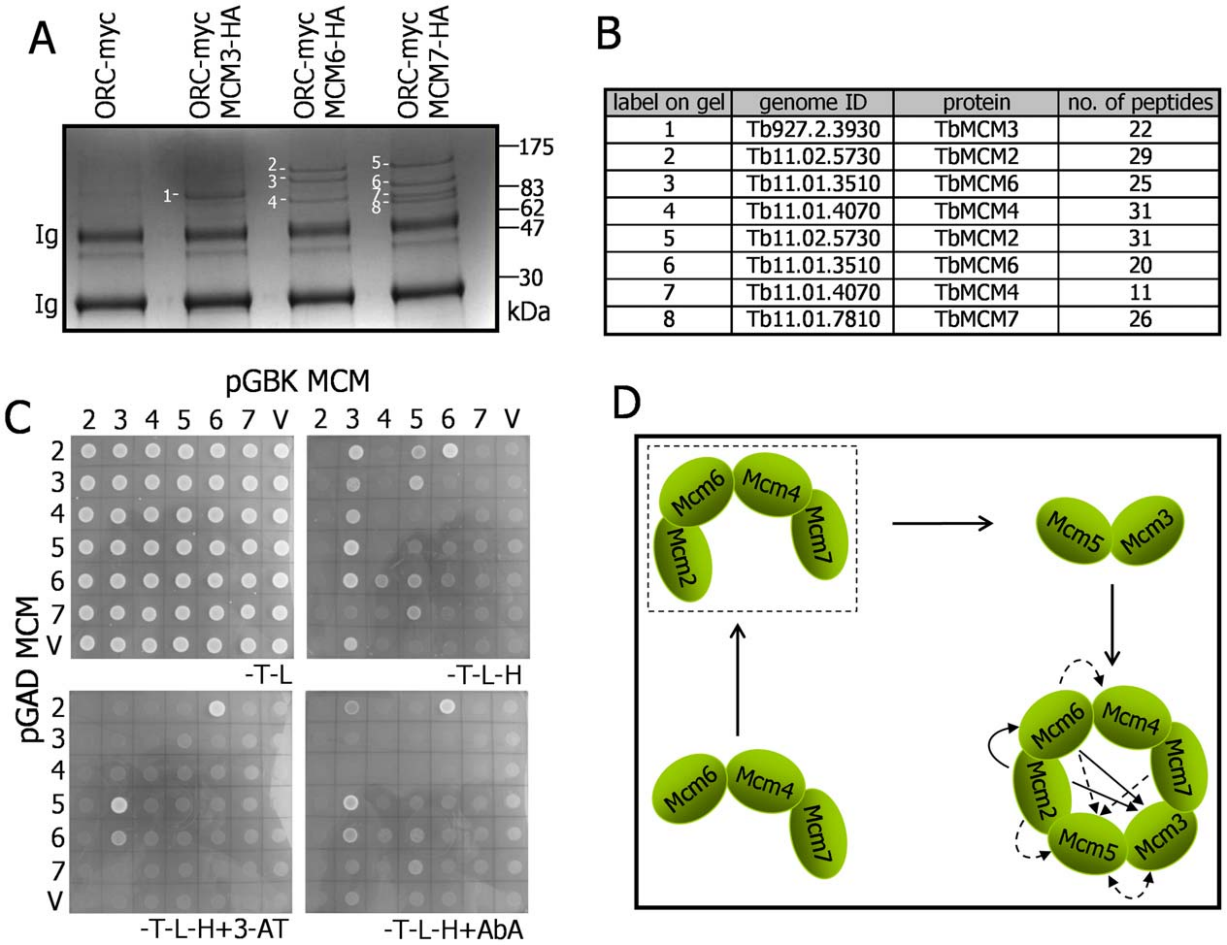
Mass spectrometric characterisation of *T. brucei* TbMCM-HA immunoprecipitates and yeast two hybrid analysis reveals MCM subunit interactions. **A.** Eluates are shown from immunoprecipitations (IP) using anti-HA antibody from *T. brucei* cell lines co-expressing TbORC1/CDC6-Myc and TbMCM3-HA, TbMCM-6HA or TbMCM7-HA; as a control an IP eluate from a cell expressing TbORC1/CDC6-Myc, but no HA-tagged protein, is also shown. Proteins in the IP eluates were separated by SDS-PAGE and visualised by colloidal commassie staining; size markers are shown and Ig indicates immunoglobulin polypeptides. Bands that were excised and analysed by mass spectrometry are numbered; the results of this analysis are shown in **B,** where the number of unique peptides identified for each band is shown, as well as the *T. brucei* gene ID and MCM subunit. **C.** Inter-MCM subunit interactions were examined by yeast 2 hybrid analysis. Growth of yeast clones co-expressing individual TbMCM subunits (numbered 2–7, indicating TbMCM2–7) as fusions with the Gal DNA binding domain (pGBK-MCM) and with the Gal activating domain (pGAD-MCM) is shown; as a control, growth of the fusion protein-expressing plasmids are shown when co-transformed with pGBK or pGAD vectors without any *MCM* gene insert (V). Growth on minimal medium lacking tryptophan, leucine and histidine (-T-L-H) indicates weak interaction, while growth on the same media supplemented with Aureobasidin A (-T-L-H+AbA) or 3′ aminotriazole (-T-L-L+3-AT), indicates strong interaction; growth on medium lacking only tryptophan and lecuine (-T-L) shows that the cells that cannot grow through interaction are viable. **D** shows a model for the assembly and subunit architecture of the MCM hexamer in eukaryotes; a putative subunit complex identified by IP in this analysis is indicated (dashed box), while intersubunit interactions revealed by yeast 2 hybrid analysis are shown in the putative heterohexamer (single- and doubled-headed arrows denote one- and bi-directional interactions, respectively, and strong and weak interactions are distinguished by solid and dashed lines, respectively).

To identify TbMCM subunit interaction partners, we performed immunoprecipitation (IP) of HA-tagged TbMCM3, TbMCM6 and TbMCM7 with anti-HA antiserum and separated the IP eluates on an SDS-PAGE gel. Colloidal Coomassie staining revealed distinct patterns of bands for TbMCM7-HA (4 bands), TbMCM6-HA (3 bands) and TbMCM3-HA (1 band) that were absent in an anti-HA IP control from the TbORC1/CDC6-Myc cells (Fig. 2). Each band was excised and protein fingerprinted by Liquid Chromatography-Electrospray Tandem Mass Spectrometry (LC-ES MS/MS). The resulting MS/MS spectra were used to interrogate the *T. brucei* genome database (TritrypDB) using MASCOT software and each band yielded at least 11 unique peptides (MASCOT score of greater than 30; *p*<0.05) that confidently matched a single ORF; all were MCM proteins. These data show the following: IP of TbMCM6-HA coIPs TbMCM2 and TbMCM4; IP of TbMCM7-HA co-IPs TbMCM2, TbMCM4 and TbMCM6; and the single band excised from the TbMCM3-HA IP was TbMCM3 itself. Thus, we find that a subcomplex can be detected containing TbMCM2, TbMCM4, TbMCM6 and TbMCM7 in PCF whole cell extracts in the absence of cross-linking. Such an MCM sub-complex has been described in several eukaryote species [4], suggesting that this aspect of TbMCM helicase structure is conserved. We did not, however, detect interaction between the above subcomplex and TbMCM3 or TbMCM5, nor did IP of TbMCM3-HA reveal interaction with TbMCM5. This approach also did not reveal co-IP of TbORC1/CDC6 and any of the TbMCM subunits analysed.

To probe further the interactions between *T. brucei* MCM subunits, we used yeast 2-hybrid analysis, co-expressing pairwise combinations of the six proteins as both ‘bait’ and ‘prey’ (Fig. 2C; more detailed analysis in Fig. S1). In contrast to the extensive intersubunit interactions observed for human Mcm proteins [47] in such analysis, including between each putative adjacent MCM subunit in the hexamer, we detect more limited interactions (summarised in Fig. 2D). Nevertheless, this analysis suggests that TbMCM3 and TbMCM5, which are thought to form a subcomplex that was not detected by IP, can interact, and that both of these subunits can interact with two further subunits of the putative TbMCM2/6/4/7 subcomplex. Taken together, these data are compatible with the order of MCM subunits that has been proposed for the eukaryotic replicative helicase heterohexamer [4].

### Testing for interaction between TbORC1/CDC6 and TbMCM

**Figure 3:**
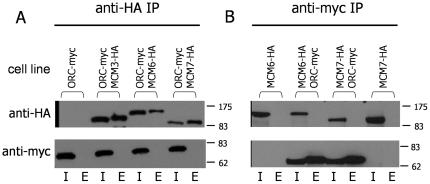
Western blot analysis of TbORC1/CDC6-Myc and TbMCM-HA immunoprecipitations. **A.** Input (I) and eluate (E) samples from immunoprecipitations (IPs) from procyclic form whole cell extracts using antibody against HA are shown from cells co-expressing TbORC1/CDC6-Myc (ORC-myc) and TbMCM3-HA, TbMCM6-HA or TbMCM7-HA, as well as from control cells expressing only Myc-tagged TbORC1/CDC6. Samples were separated on a 10% SDS-PAGE gel, transferred to a membrane and probed with anti-HA antibody (upper panel) or with anti-Myc antibody (lower panel). **B** shows the reciprocal experiment in which IP was performed with anti-Myc antibody from cells co-expressing TbORC1/CDC6-Myc and TbMCM6-HA or TbMCM7-HA, and from control cells expressing only HA-tagged MCM6 or MCM7. Size markers (kDa) are indicated.

To test more directly for TbORC1/CDC6 and TbMCM interactions we next used anti-HA antiserum to precipitate TbMCM3-HA, TbMCM6-HA or TbMCM7-HA from whole cell extracts of the double-tagged cells co-expressing TbORC1/CDC6-Myc. Probing the inputs and eluates with anti-HA antibody showed that each TbMCM subunit could be recovered by IP from the double tagged cells, but not from a control cell expressing TbORC1/CDC6-Myc but not expressing an HA-tagged TbMCM subunit (Fig. 3A). Probing the same samples with anti-myc antibody failed to reveal evidence for interaction between any of the three TbMCM subunits analysed and TbORC1/CDC6-Myc, which was clearly detectable in the input samples.

Since TbMCM might only interact with chromatin-bound TbORC1/CDC6 at replication origins, it is possible that a substantial portion of the cellular pool of TbMCM may be unbound to DNA/TbORC1/CDC6, masking any interactions from being detected by IP of an MCM subunit. We therefore carried out the reciprocal IP, using anti-Myc antibody to recover TbORC1/CDC6-Myc from cell extracts. To do this, we generated cells expressing only TbMCM6-HA or TbMCM7-HA (Fig. 3B), providing controls for TbMCM6-HA, TbORC1/CDC6-Myc and TbMCM7-HA, TbORC1/CDC6-Myc double expressers. Probing the input and eluate samples from the four cells showed that anti-Myc IP recovered a band of the expected size for TbORC1/CDC6-Myc from both double-tagged cells that was, as expected, absent from the TbMCM-HA control cells. However, immunoblotting for TbMCM6-HA or TbMCM7-HA using anti-HA antibody did not detect the proteins in the eluates from any of the IPs (Fig. 3B), suggesting that interaction with TbORC1/CDC6-Myc is undetectable in these conditions.

### Identification of three novel ORC1/CDC6-interacting factors

Having shown that it is possible to efficiently IP TbORC1/CDC6-Myc from *T. brucei* PCF cell extracts, we next asked if we could identify any factors with which the protein interacts. To do this, whole cell extracts were prepared from PCF TbORC1/CDC6-Myc cells and from untagged (TREU927 wild type, PCF) cells and subjected to IP. Eluates from each IP were separated by 10% SDS-PAGE, which revealed a large number of bands in each, with no obvious differences by visual inspection (data not shown). Proteins throughout the gel lanes, from each IP, were excised and analysed by LC-ES MS/MS and the resulting MS/MS spectra were used to interrogate the *T. brucei* genome (TritrypDB). These data were first filtered by excluding all individual proteins that were common to both IP eluates, and further filtered by excluding proteins that were only present in the TbORC1/CDC6-myc eluate but where proteins that they were likely to functionally interact with were present in the control IP eluate (such as ribosomal proteins or translation factors), or with clear non-nuclear functions. We then ranked the resulting TbORC1/CDC6-Myc IP-specific proteins according to the number of unique peptide hits/protein observed in the MS/MS. TbORC1/CDC6 was thus revealed (10 unique peptides), validating the approach. To further limit our analysis, we considered only proteins recognised by >3 peptides, all of which have not been ascribed potential functions experimentally or by sequence homology. For each polypeptide, position-specific iterative (psi) BLAST, Protein Homology/analogY Recognition Engine (PHYRE) and PFAM searches were carried out. This revealed three proteins that could potentially be related to eukaryotic ORC subunits, or Cdc6 (see below), and were therefore analysed further: Tb09.160.3120 (10 peptides), Tb927.10.13380 (10 peptides) and Tb927.10.7980 (4 peptides). To test whether these proteins interact with TbORC1/CDC6, constructs were generated that allow each to be expressed as a C-terminal fusion with six copies of the HA epitope, after integration into the endogenous loci. The constructs were transformed into the TbORC1/CDC6-Myc tagged PCF line cell line and clones selected in which expression of the tagged proteins was validated by westerns (data not shown). As controls, the constructs were also transformed into WT PCF cells and clones expressing each HA-tagged protein were similarly validated (data not shown).

**Figure 4:**
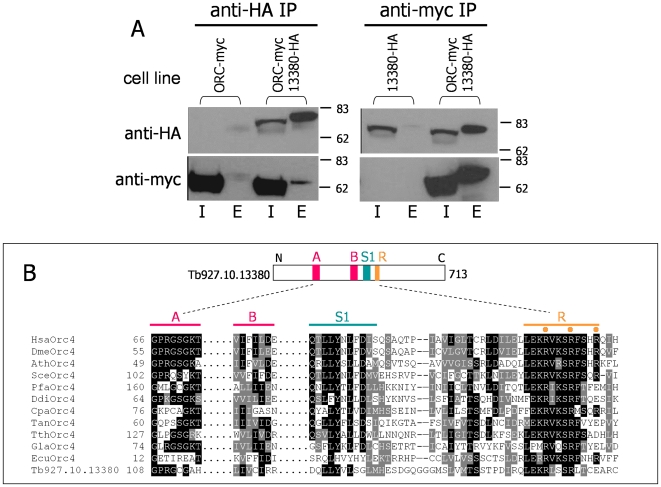
Identification of a *T. brucei* ORC1/CDC6-interacting protein as a putative orthologue of eukaryotic Orc4. **A.** Input (I) and eluate (E) samples from immunoprecipitations (IPs) from procyclic form whole cell extracts are shown using antibody against HA (anti-HA) or against Myc (anti-Myc). Anti-HA IP was performed from cells co-expressing TbORC1/CDC6-Myc (ORC-myc) and Tb13380-HA, or from control cells expressing only Myc-tagged TbORC1/CDC6; anti-Myc IP was from cells co-expressing TbORC1/CDC6-Myc and Tb13380-HA, or from control cells expressing only HA-tagged Tb13380. In all cases IP samples were separated on a 10% SDS-PAGE gel, transferred to a nylon membrane and probed with anti-HA antibody (upper panel) or with anti-Myc antibody (lower panel). Size markers (kDa) are indicated. **B** A sequence comparison of the predicted Tb13380 polypeptide (translation of *T. brucei* gene ID Tb927.10.13380) with Orc4 proteins from a number of eukaryotes (black and grey boxing highlights residues identical or conserved, respectively, in 50% of the sequences). For the following species Orc4 has been functionally or bioinformatically identified: *H. sapiens* (Hsa, O43929), *D. melanogaster* (Dme, AAF47276.1), *A. thaliana* (Ath, CAE01428), *S. cerevisiae* (Sce, P54791), and *T. thermophila* (Tth, 51.m00235). Also shown are putative Orc4 orthologues from further species: *P. falciparum* (Pfa, PF13_0189), *Dictyostelium discoideum* (Ddi, DDB0168430), *Cryptosporidium parvum* (Cpa, cgd2_1550), *Theileria annulata* (Tan, TA12985), *Giardia lamblia* (Gla, ctg02_3) and *Encephalitozoon cuniculi* (Ecu, NP_59761). The Tb13380 (ORC4) polypeptide is shown diagrammatically (number of amino acid residues is indicated), highlighting regions of conservation around motifs involved in nucleotide binding and hydrolysis: Walker A and B boxes (A and B, red boxes), an Arginine finger (R, orange box) and a Sensor 1 motif (S1, green boxes).

IP analysis of Tb927.10.13380 (Tb13380) is shown in Fig. 4A. Anti-HA antiserum was used first to IP Tb13380-HA from whole cell extracts of double-tagged cells co-expressing TbORC1/CDC6-Myc and Tb13380-HA, and from a control cell expressing only myc-tagged TbORC1/CDC6. Probing western blots of the inputs and elutes from the IPs with anti-HA antibody showed that Tb13380-HA was recovered by IP from the double tagged cells, but not from the control. Probing the same samples with anti-myc showed that TbORC1/CDC6-Myc was also recovered, indicating interaction. The reciprocal experiment confirmed this interaction: anti-myc antiserum was able to IP TbORC1/CDC6-Myc from the Tb13380HA, TbORC1/CDC6Myc double expresser cell, and Tb13380-HA was also recovered. The same IP did not recover Tb13380-HA from the single expresser control.

Second iteration psiBLAST searches of the NCBI database using the Tb13380-predicted polypeptide sequence (713 amino acid residues) identified *Drosophila melanogaster* (Dme)Orc4 (E-value, 9e-14), and then Orc4 subunits from further organisms at lower, yet highly significant, expectancy values (0.003≤E-value≤1e-13). In the reciprocal BLAST search, using DmeOrc4 sequence to query TritrypDB, the ‘top’ hit (E-value, 0.0021) was Tb13380. To examine if Tb13380 displays broader sequence homology with eukaryotic Orc4 subunits, the complete predicted sequence of Tb13380 was aligned with a range of Orc4 polypeptides, both from organisms in which the protein has been functionally characterised and from other organisms (primarily protists) where we could recover putative ORC4 homologues by database searches (Fig. 4B). Overall, Tb13380 displays limited amino acid sequence homology with Orc4 subunits (for example, 33% similarity and 16% identity with DmeOrc4, and 33% similarity and 15% identity with *Arabidopsis thaliana* Orc4). However, in phylogenetic trees Tb13380 groups robustly with Orc4, when all other ORC subunits are considered (Fig. S2). We conclude that Tb927.10.13380 encodes a divergent orthologue of the Orc4 subunit of eukaryotic ORC. Homology with Walker A and B boxes, and a sensor 1 motif, found within AAA+ ATPase proteins [9], [12] can be identified. However, it is not clear that TbORC4, and indeed a number of the other putative protistan Orc4 homologues, are functional NTPases, since they lack critical, conserved residues. TbORC4 lacks a crucial lysine residue (K62 of DmeOrc4; K108 of *S. cerevisiae* Orc4; K114A in Tb13380) in the Walker A motif, which binds the nucleotide, and the Walker B motif, which binds Mg++, appears degenerate. A further important motif (arginine finger, or box VII) possesses a key arginine residue proposed to interact with the γ-phosphate of the adenine nucleotide [9], [48]. This interaction is needed to coordinate ATP hydrolysis with a conformational change, a critical feature of protein-protein interactions [48]. The arginine finger, and surrounding sequence, appears to be well conserved between TbORC4 and other eukaryotic Orc4 proteins. TbORC4 is conserved and syntenic across the sequenced kinetoplastids: orthologues, annotated as hypothetical proteins, can be found in *T. cruzi* (Tc00.1047053506357.20 and Tc00.1047053511277.92, 54% identity with Tb13380) and *L. major* (LmjF18.0720, 38% identity with Tb13380).

**Figure 5:**
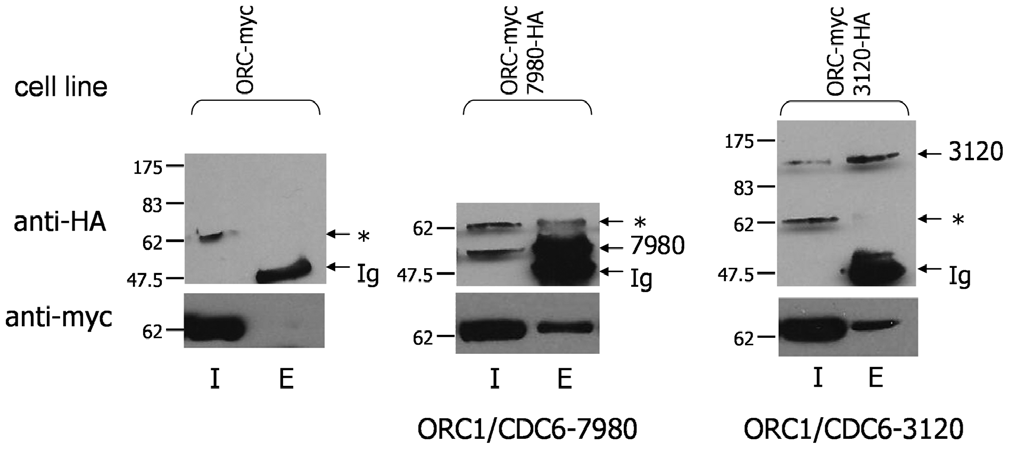
Co-immunoprecipitation demonstrates interaction between TbORC1/CDC6 and two novel factors. Input (I) and eluate (E) samples from immunoprecipitations (IPs) using antibody against HA (anti-HA) are shown from procyclic *T. brucei* whole cell extracts of cells co-expressing TbORC1/CDC6-Myc (ORC-myc) and either Tb7980-HA (IP labelled ORC1/CDC6-7980) or Tb3120-HA (IP labelled ORC1/CDC6-3120); a control anti-HA IP is shown from cells expressing only Myc-tagged TbORC1/CDC6. Samples were separated by SDS-PAGE, transferred to a nylon membrane and probed with anti-HA antibody (upper panel) or with anti-Myc antibody (lower panel). Bands corresponding with immunoglobulin heavy chain (Ig), an anti-HA cross-reacting band (*) and with either HA-tagged Tb7980 or Tb3120 are indicated in the HA IP samples; size markers (kDa) are indicated.

Analysis of TbORC1/CDC6 interaction with Tb09.10.3120 (Tb3120) and Tb927.10.7980 (Tb7980) is shown in Fig. 5. Anti-HA antibody was used to IP Tb7980-HA from a TbORC1/CDC6-Myc, Tb7980-HA double expresser cell and Tb3120-HA from a TbORC1/CDC6-myc, Tb3120-HA double expresser; IP was also performed with anti-HA antibody from a control TbORC1/CDC6-Myc single expresser cell. For Tb3120, probing with anti-HA antibody showed that the protein was efficiently recovered by IP from the double expresser cells, but not from the control. It was not possible to evaluate fully the success of the IP of Tb7980-HA from the double expresser, because Tb7980-HA nearly co-migrates with the IgG heavy chain fragment in the antibody used for the IP, and this is seen in the western blot strategy adopted here. Nevertheless, for both IPs, the anti-Myc blot revealed a band of the expected size for TbORC1/CDC6-Myc in the eluate from the TbORC1/CDC6-Myc, Tb7980-HA and TbORC1/CDC6-Myc, Tb3120-HA double expressers, which was not seen in the controls, indicating interaction.

In contrast to the strong bioinformatic evidence for identification of TbORC4 (above), similar homology-based analyses give few clues as to the potential functions of Tb7980 and Tb3120, which may be kinetoplastid-specific. Tb7980 is predicted to be 441 amino acids in length and is conserved and syntenic across the sequenced kinetoplastids (Tc00.1047053506247.280 in *T. cruzi*, 66% identity; LmjF36.6700 in *L. major*, 44% identity). The strongest evidence that this protein may be ORC-like comes from PHYRE searches, which suggest structural similarity with archaeal Orc1/Cdc6 proteins: e.g. Cdc6p from *Pyrobaculum aerophilum* (80% precision) and Orc1/Cdc6 from *Sulfolobus Solfataricus* (80% precision). A putative Walker A motif (GPPGSGKT; residues 33–40) is present in the polypeptide sequence. Though further motifs conserved in AAA^+^ ATPases [9] could not be identified, PFAM domain analysis suggested that the protein has similarity with this enzyme family (data not shown). However, despite these ORC-like features, we cannot meaningfully align the sequence of Tb7980 with Orc1 and/or Cdc6 from eukaryotes or from archaea, or with Orcs2–6. Tb3120 appears yet more diverged. It is predicted to be 1018 amino acids in length, and has syntenic orthologues in *T. cruzi* and *L. major* (Tc00.1047053511585.90 in *T. cruzi*, 48% identity; LmjF01.0660 in *L. major*, 24% identity). PHYRE searches provide only very weak evidence for putative structural similarity with archaeal Orc1/Cdc6 proteins (data not shown), and we cannot identify AAA^+^ ATPase motifs that are characteristic of ORC-related factors. As for Tb7980, homologous proteins could not be identified in eukaryotes beyond the kinetoplastids, or in archaea, by BLAST searches.

### RNAi of all three ORC1/CDC6-interacting factors results in growth defects in procyclic form *T. brucei*

**Figure 6:**
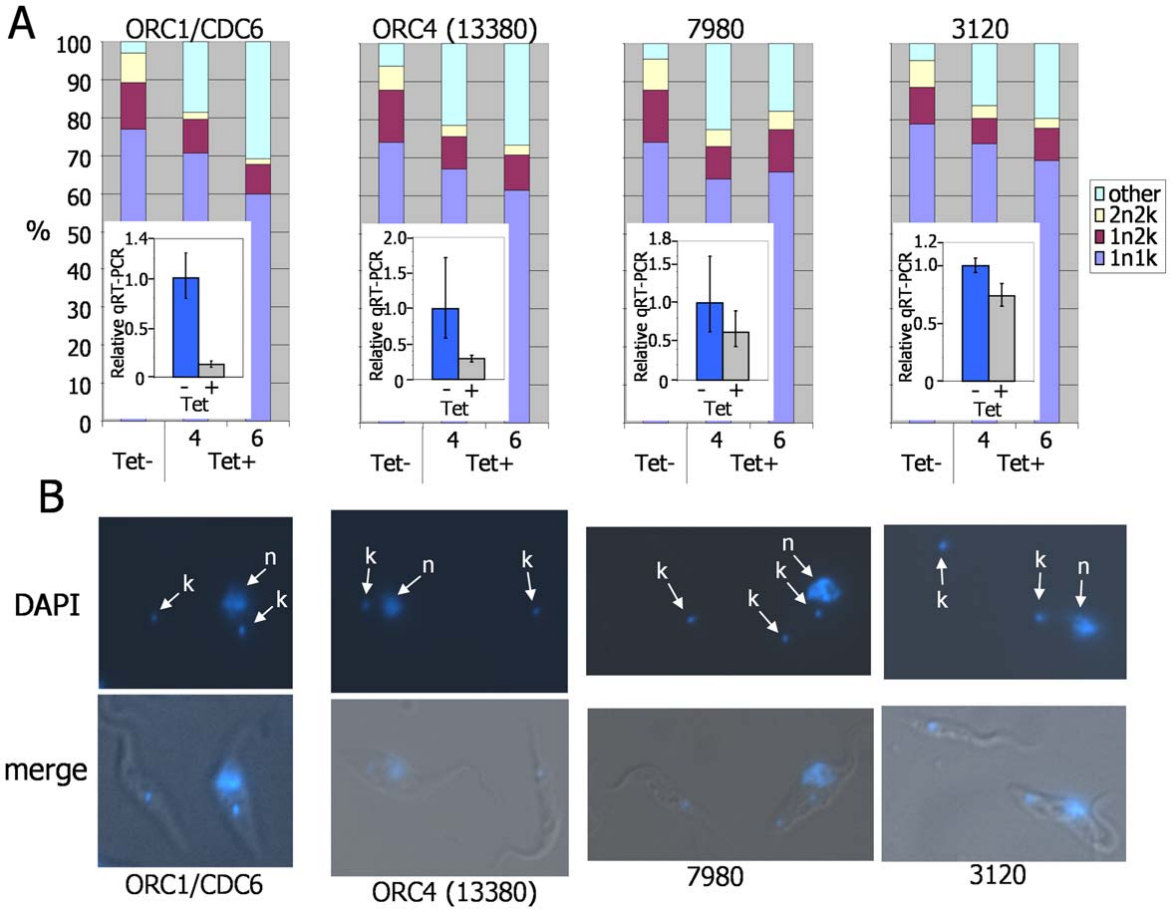
Effect of TbORC1/CDC6, TbORC4, Tb7980 and Tb3120 RNAi on procyclic form *T. brucei*. **A.** Analysis of nuclear (N) and kinetoplast (K) DNA configurations in procyclic form *T. brucei* cells 4 and 6 days post-RNAi induction (induced by tetracycline; Tet+) against *TbORC1/CDC6*, *TbORC4 (13380)*, *Tb7980 and Tb3120*; for each gene, N and K configurations are also shown in cells without RNAi induction (Tet−). Graphs depict the proportion of cells (derived by counting >200 DAPI-stained cells) with conventional 1N1K, 1N2K, or 2N2K configurations, or with any aberrant configuration (others). In each graph, the insert shows the extent of loss of cognate mRNA by quantitative reverse transcriptase PCR (qRT-PCR): levels of mRNA are shown four days after RNAi induction (Tet+) relative to the uninduced cells (Tet−), where qRT-PCR amplification has been set as 1.0 (values are the mean of four experimental repetitions and vertical lines denote standard deviation). **B** Representative images, showing aberrant cells with 0N1K DNA configuration seen 6 days after induction of RNAi of the gene indicated. Cells are shown with DNA stained by DAPI (N and K are arrowed), and as merge of DAPI and phase contrast images.

To test whether TbORC4 (13380), Tb7980 and Tb3120 act in nuclear DNA replication, an RNAi approach was used. For each gene, and for *TbORC1/CDC6*, we generated constructs that provide tetracycline-inducible expression of RNAi once transformed into transgenic *T. brucei* PCF cells (Lister 427 pLew13 pLew 29) [49]. For each gene, RNAi induction had no detectable effect on growth for up to 3 days (∼7–8 population doublings; data not shown), and thereafter (days 4–6) reduced but did not abolish growth (Fig. S3). These relatively minor and slow to accumulate effects on cell survival are consistent with a previous description of growth kinetics in PCF *T. brucei* following TbORC1/CDC6 RNAi (M. Klingbeil, pers.com.) [43]. Analysis of DNA content by fixing and DAPI-staining the cells, and then counting the ratio of nuclear (N) and kinetoplast (K) DNA visible in individual cells, revealed potential replication-associated defects (Fig. 6; Fig. S4). As has been described for TbORC1/CDC6 [43], and confirmed here, RNAi of each gene resulted in the accumulation of aberrant cells that do not conform to the 1N1K, 1N2K or 2N2K DNA configurations that mark the normal course of cell division in *T. brucei* [50]. In all cases, these aberrant cells were 0N1K ‘zoids’, indicating that they had lost nuclear DNA, and their accumulation was mirrored by similar reduction in 1N1K cell numbers, suggesting they arise from cytokinesis of 1N2K cells that have undergone kinetoplast replication and division but have failed to complete nuclear DNA replication. The numbers of these zoids, which never amounted to more than ∼5% of the uninduced cells, appeared to reflect the extent of mRNA loss following RNAi: TbORC1/CDC6 and TbORC4 mRNA levels were relatively strongly reduced (by ∼85% and 75%, respectively) and zoids made up 31% and 28%, respectively, of the population 6 days after RNAi; Tb7980 and Tb3120 RNAi appeared less effective (∼40% and 30% mRNA reduction, respectively) and zoids accumulated to a lesser extent (18% and 19% of the population after 6 days). Despite this pronounced accumulation of zoids, we have not to date been able to demonstrate directly a role for the putative ORC-like factors in nuclear DNA replication. To do this, we attempted to measure the extent of 5′ bromodeoxyuridine (BrdU) incorporation after RNAi by dot-blotting genomic DNA and probing with anti-BrdU monoclonal antibody [51]. For each of TbORC4, Tb7980 and Tb3120, we have been unable to detect any reduction in BrdU signal four, six or even 10 days post-RNAi induction (data not shown). However, it appears that this assay is relatively insensitive unless RNAi is strongly penetrative. When we analysed an RNAi clone that resulted in ∼90% loss of TbORC1/CDC6 mRNA, BrdU incorporation was detectably reduced after four days, and this was concomitant with loss of 4C DNA and increased amounts of S-phase and sub-2C DNA in FACS analysis of propidium iodide (PI)-stained DNA (Fig. S5), very similar to that described by Godoy et al (2009) [43]. In contrast, in another clone in which only ∼75% loss of TbORC/CDC6 mRNA could be seen, no effect on BrdU incorporation could be detected after 6 days and the perturbation of DNA content by FACS was noticeably less severe (Fig. S5). As the maximum extent of mRNA loss we have seen post-RNAi is ∼75% for ORC4 (and is substantially less for Tb7980 and Tb3120, despite screening a number of transformant clones), it seems likely that the extent of RNAi is below a threshold needed to see an effect on nuclear DNA replication by the BrdU dot blot assay adopted. As a result, despite the strikingly similar growth response of RNAi against each gene, we cannot exclude that the formation of zoids in these conditions is not a result of an effect on nuclear DNA replication but is due to perturbation of the cell cycle.

### ORC1/CDC6 and interacting factors are essential in bloodstream form *T. brucei*

**Figure 7:**
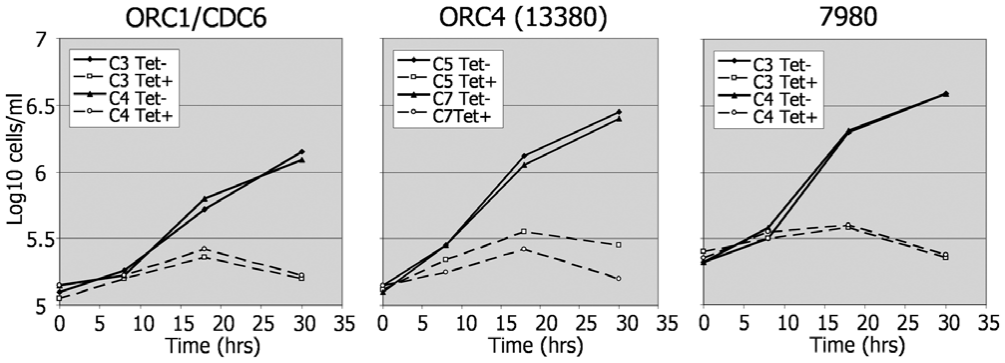
RNAi of TbORC1/CDC6, TbORC4 and Tb7980 in bloodstream form *T. brucei* cells results in rapid growth arrest. Growth curves are shown for bloodstream form *T. brucei* cells in the absence or presence of tetracycline (tet−, shown as solid line, and tet+, dashed line, respectively), which induces RNAi, targeting either *TbORC1/CDC6*, *TbORC4 (Tb13380)*, or *Tb7980* mRNA. For each factor, cell density over time was examined in two clonal RNAi cell lines (identified by C).

To date, ORC1/CDC6 function has been examined only in tsetse fly-infective PCF *T. brucei* [43](M. Klingbeil, pers.com.). To investigate the importance of ORC1/CDC6 and its interacting factors in mammal-infective *T. brucei*, we perfomed RNAi in bloodstream form (BSF) cells. For this the same RNAi constructs used in PCF cells (above) were transformed into an established transgenic *T. brucei* BSF cell line (Lister 427 pLew13 pLew90) [52] that allows tetracycline-inducible, gene-specific RNAi. For each of *TbORC1/CDC6*, *TbORC4* and *Tb7980* two independent transformant clones were selected and growth was followed in the absence or presence of tetracycline (Fig. 7). Induction of RNAi resulted in strikingly similar, severe growth defects for each gene. In the case of ORC4 knock down, a reduction in growth was observed after ∼8 hrs (essentially a single population doubling for such cells in culture) relative to the uninduced controls. In all three knock-downs, by 18–30 hrs post-induction of RNAi the cell concentrations dropped, indicating cell death, and thereafter viable cells became virtually impossible to count in these conditions. These results suggest that each gene is essential for viability of BSF *T. brucei* cells. For *Tb3120* we have not yet identified a BSF transformant clone in which RNAi could be demonstrated to have occurred, so this gene was excluded from further analysis.

**Figure 8:**
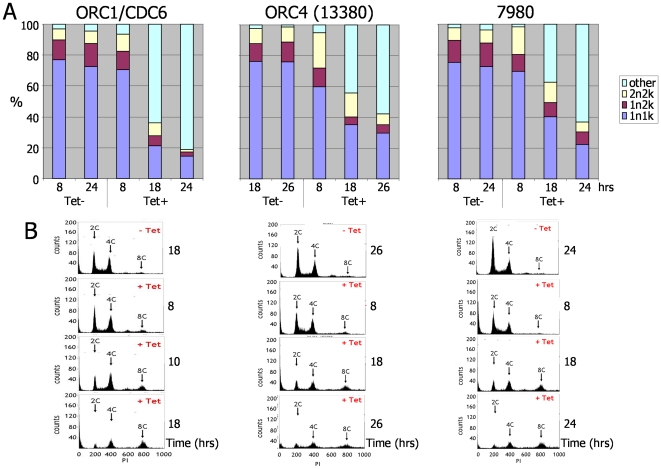
Effect of TbORC1/CDC6, TbORC4 and Tb7980 RNAi on bloodstream form *T. brucei*. **A.** Analysis of nuclear (N) and kinetoplast (K) DNA configurations in bloodstream form *T. brucei* cells at time points following RNAi induction (induced by tetracycline; Tet+) against *TbORC1/CDC6*, *TbORC4 (13380)* and *Tb7980*; for comparison, N and K configurations are shown in cells without RNAi induction (Tet−). Graphs depict the proportion of cells (derived by counting >200 DAPI-stained cells) with conventional 1N1K, 1N2K, or 2N2K configurations, or with any aberrant configuration (grouped as others). **B.** FACS profiles of propidium iodide (PI)-stained cells after RNAi induction (Tet+) are shown as histograms after FACS sorting, sampled at the time points post-induction (control cells, without RNAi induction (Tet−), are shown sampled at the time shown, corresponding to growth from an equivalent starting density to the RNAi- induced cells). Peaks corresponding with cells containing 2C and 4C DNA content are indicated, as is the peak position for cells with 8C content (C represents haploid DNA content).

To determine whether the growth defects observed upon RNAi induction correlate with a cell cycle defect, DNA content was monitored by DAPI-staining the cells as described above (Fig. 8A). The results for each gene were again strikingly similar. After RNAi depletion of any of the three genes, an increase in cells with >2N>2K DNA configuration was apparent (Fig. S6), rising from ∼2% of the population at 8 hrs post-RNAi induction to approximately 60% at 24–26 hrs post induction. In many cases in these aberrant cells it was impossible to determine accurately N and K numbers. Concomitant with this was a decrease in 1N1K cells, reducing from ∼70% to ∼20% within the same time period. This cell morphological defect was not observed in any control (uninduced) population, where 1N1K cells constituted ∼80% of the population between 8 and 26 hrs, and aberrant cells never amounted to more than ∼3% of any population. To further characterise the effect of depleting each protein on *T. brucei* DNA content, FACS was used to examine PI-stained DNA from RNAi-induced versus non-induced cells for up to 26 hrs (Fig. 8B). The resulting FACS profiles showed that RNAi in each case resulted in a decrease in the proportion of cells with 2C and 4C DNA content (the former being depleted more rapidly), with a concomitant increase in the proportion of cells with 8C DNA content. The increase in the 8C cells appears consistent with the accumulation of multinucleate cells observed in DAPI staining (Fig. 8A).

## Discussion

**Figure 9:**
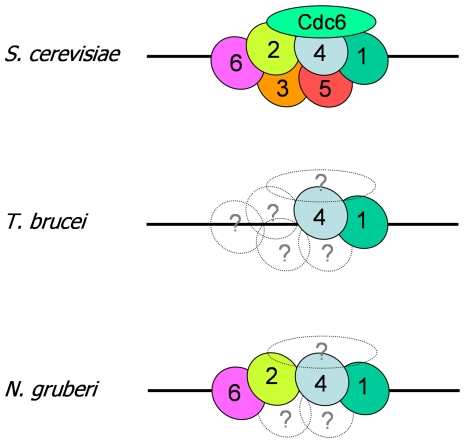
Origin Recognition Complex architecture in the eukaryotes *S. cerevisiae*, *T. brucei* and *N. gruberi*. The architecture of the Origin Recognition Complex (ORC; composed of Orc subunits numbered 1–6), bound to the Orc1-related factor Cdc6 and to DNA (black line), is shown for *S. cerevisiae* based on work by Chen et al [22]; the specific arrangement of Orcs 2–5 is inferred from Moreno del-Alamo [58]. In *T. brucei*, recognisable ORC subunit orthologues are identified, while subunits that are absent or highly diverged are shown as dotted circles containing question marks. The *T. brucei* ORC subunit indicated as Orc1 appears to be a bi-functional Orc1-Cdc6 protein, and it is unknown if it therefore occupies a distinct architectural position in the ORC or adopts a distinct structure. Putative ORC subunits identified bioinformatically in *N. gruberi*, a free-living relative of *T. brucei*, are shown for comparison; here again, Orc1 appears to be an Orc1-Cdc6 fusion.

In most eukaryotes examined to date, ORC has a conserved architecture, composed of six subunits, Orcs1–6. Of these, Orcs1–5 belong to the AAA+ ATPase family, which also contains the Orc1-related factor Cdc6 [7], [9], [12,12], while Orc6 is less conserved and appears not to be an AAA+ ATPase. Archaea, in contrast, lack Orcs2–6 and instead possess one or more paralogues of a protein, Orc1/Cdc6, that may fulfil both ORC and Cdc6 functions [2]. Previous work, based on bioinformatic analyses, has suggested that *T. brucei* and related kinetoplastid parasites may possess a simple archaeal-like system of a single origin designation factor [42], [43], and the single identified ORC-related protein has been named ORC1/CDC6 to reflect this. Here, we have searched for factors that interact with *T. brucei* ORC1/CDC6 in PCF (tsetse fly infective) cells and identified three, each conserved in all kinetoplastids. Of these, one is clearly a further ORC-like factor, most closely related to eukaryotic Orc4 and very likely to be a *T. brucei* orthologue whose sequence is sufficiently diverged to have escaped detection by similarity searches. The two other proteins, though displaying sequence features that suggest they may be ORC-related, are so diverged that we cannot assign orthology. A further TbORC1/CDC6-interacting factor, TbORC1b, has been described [45], which was not recovered in the IP approach we adopted and also has no clear ORC subunit orthology. The identification of TbORC4 indicates that ORC in *T. brucei* is closer to the eukaryotic paradigm than previously thought (Fig. 9). However, as we cannot identify orthologues of Orcs 2, 3, 5 or 6, ORC may yet be highly diverged.

Three evolutionary scenarios might explain *T. brucei* ORC divergence. First, Orc2, Orc3, Orc5 and Orc6 are truly absent and this is because ORC structure in *T. brucei* reflects a simplified, ancestral state of this machinery [21], [43]. Alternatively, TbORC1/CDC6 and TbORC4 may be the only survivors of streamlining of the six-component ORC machinery, perhaps reflecting the generalised demands of a parasitic lifestyle [36]. Finally, it is possible that all ORC subunits are present in *T. brucei* but most have become highly diverged due to lineage-specific demands on DNA replication (for instance, constraints on ORC recruitment in a genome in which gene expression is nearly exclusively polycistronic and mRNA abundance controlled post-transcriptionally) [53]. Searching the sequenced genomes of a wide range of protistan organisms provides some insight into this (Table 1; Text S1). The absence of clear homologues of each of the six ORC subunits is not uncommon, and the presence of a single protein related to both Orc1 and Cdc6 is not limited to kinetoplastids [54]. Within the Opisthokont supergoup, six ORC proteins and Cdc6 as a separate protein are found in yeasts and metazoans, and the same factor complement is seen in the social amoeba *Dictyostelium discoidium*. However, in another Opisthokont, *Encephalitozoon cuniculi*, and in *Entamoeba histolytica*, an Amoebazoan related to *D. discoidium*, fewer factors could be identified. *E. cuniculi* is an intracellular microsporidian parasite, related to fungi such as yeast, and considerable evidence suggests *E. cuniculi* is undergoing a process of genome reduction [55], [56]. Here, then, the absence of ORC components may be genuine and reflect relatively recent evolutionary loss, illustrating that eukaryotic ORC function can be adapted to a streamlined version lacking some components. In the Excavata supergroup, which includes kinetoplastids, Orc1 and Cdc6 were nearly always identified bioinformatically as a single protein (only in *Trichomonas vaginalis* were both Orc1 and Cdc6 found) [57], and in no sequenced genome could all six ORC components be found. However, the repertoire of genes that were found was not consistent in each organism and, perhaps tellingly, the genome of *Naegleria gruberi* possessed the highest number of identifiable ORC-related factors [36]. Though it is unclear whether the Excavata is a legitimate evolutionary grouping, *N. gruberi* is to date the only free-living protist in this group whose genome has been sequenced. Fig. 9 compares putative conserved ORC factors in *N. gruberi* and *T. brucei*. The *N. gruberi* factors, like those of *T. brucei*, are highly diverged (data not shown), but appear most related to Orc1, Orc4, Orc2 and Orc6, based on BLAST searches and sequence alignments [36](data not shown). This argues that ORC architecture in *T. brucei* and kinetoplastids, like that in *E. cuniculi*, is not ancestral but reflects either evolutionary divergence or loss of the constituent proteins. The latter scenario would be consistent with a much broader evolutionary selection for genome compaction in these parasites [36].

**Table 1:** A comparison of the presence (+), absence (−) or number of detectable ORC subunit and Cdc6 proteins encoded by the genomes of a range of eukaryotic species, which are grouped into taxa and into five supergroups; ‘+’ in parenthesis indicates that a single protein is found that is related to both Orc1 and Cdc6.

Supergroup	Taxon	Organism	Orc1	Orc2	Orc3	Orc4	Orc5	Orc6	Cdc6
Excavata^a^	Diplomonadida	*G. lamblia*	(+)	−	−	+	−	−	(+)
	Euglenozoa	*T. brucei*	(+)	−	−	+	−	−	(+)
		*T. cruzi*	(+)	−	−	+	−	−	(+)
		*L. major*	(+)	−	−	+	−	−	(+)
	Heterolobosea	*N. gruberi*	(+)	+	−	+	−	+	(+)
	Parabasala	*T.vaginalis*	+	+	−	+	−	−	+
Chromalveolata	Stramenopile	*T. pseudonana*	+	+	−	−	−	−	+(2)
	Apicomplexa	*T. annulata*	+	+	−	+	−	−	+
		*P. falciparum*	+	+	−	+	+	−	+
		*C. parvum*	+	+	−	+	+	−	+
	Ciliophora	*T. thermophila*	+	+	−	+	+	−	+
Plantae	Viridiplantae	*A. thaliana*	+	+	+	+	+	+	+
Amoebozoa	Entamoebidae	*E. histolytica*	+	−	−	−	−	−	+
	Mycetozoa	*D. discoideum*	+	+	+	+	+	+	+
Opisthokonta	Microsporidia	*E. cuniculi*	+	+	−	+	−	−	+
	Fungi	*S. pombe*	+	+	+	+	+	+	+
		*S. cerevisiae*	+	+	+	+	+	+	+
	Metazoa	*D. melanogaster*	+	+	+	+	+	+	+
		*H. sapiens*	+	+	+	+	+	+	+

a the supergroup Excavata is often split into two supergroups, one containing Euglenozoan, Heterolobosean and Jackobid (not shown) organisms, and the other containing Parabasalid, Diplomonad and Oxymonad (not shown) organisms.

Irrespective of the arguments for and against the presence or absence of ORC subunits in extant eukaryotes, including *T. brucei*, it is clear that the most commonly conserved proteins are Orc1 and/or Cdc6 and Orc4 (Table 1). Only in two of the organisms that we have analysed can Orc4 not be identified; in most cases it is readily identified by sequence homology, and we show here that it is present in *T. brucei*, *T. cruzi* and *L. major*. Orc1 and Cdc6 are found in all organisms, albeit in some cases as one indistinguishable protein (see below). These data suggest that these are the core factors of the ORC machinery: the most resistant to loss during genome streamlining, or to change is sequence due to evolutionary pressures on ORC function. This suggests that these are central to the functioning of ORC, which has experimental support. The structure of eukaryotic ORC has not been determined to high resolution, but electron microscopy has allowed the architecture of ORC and the Orc1–5 subcomplex to be determined at low resolution in *S. cerevisiae* and *D. melanogaster* [18], [22]. The inferred architecture (Fig. 9) suggests that Orc4 and Orc1 are adjacent in the structure, with Orc4 positioned centrally and contacting Orc5 and Orc2 [58]. Functional interaction is also seen in stimulation of Orc1's ATPase by the arginine finger of Orc4, a co-operative activity needed for pre-RC function [59]. Association of Orc1 and Orc4 is also seen at *S. cerevisaie* ARS origins, where they bind near A elements, distinct from Orc2 binding around B1 elements [60]. In *S. pombe*, Orc4 appears to have assumed a yet more central role, since it has an N-terminal extension containing nine AT-hook DNA binding motifs that are needed for assembly of ORC at origins [23], [61]–[63]. That Orc4 is key to ORC association with DNA is seen by the finding that it is the ORC subunit that remains most strongly associated with chromatin after salt extraction of cell extracts [64]. Finally, during assembly and disassembly of human ORC on DNA, Orc4 has been shown to be a central mediator [17], [65]: ATP binding to Orc4 is needed to allow interaction of the subunit with an Orc2/3/5 sub-complex, which then recruits Orc1 and binds DNA; disassembly of ORC may be mediated by Orc4 ATP hydrolysis after ubiquitin-mediated proteolysis of Orc1.

Given the above, is it possible that ORC in *T. brucei* consists only of TbORC4 and TbORC1/CDC6, or are other ORC subunits present but so diverged that they have escaped detection? The predicted *T. brucei* ORC4 polypeptide may not to be a functional ATPase, as it has degenerate Walker A and B boxes (Fig. 4). In most eukaryotes Orc4 and Orc5 are highly conserved ATPases, while Orc2 and Orc3 are less conserved but are proposed to have AAA+ ATPase structure [7], [12]. It is thought that ATP binding and hydrolysis by the ORC subunits are needed for conformational changes associated with ORC assembly, DNA binding, interaction with MCM in the pre-RC, and disassembly [12], [15]–[18]. In this regard it may be significant that AAA+ proteins function as multimeric assemblies, with the ATPase active sites formed at the interface of protomers. Thus, each protein has a *cis* face (containing Walker A and B residues) and a *trans* face that juxtaposes to the *cis* face of an adjacent protomer. Interestingly, the *trans* residues of the *T. brucei* ORC4 appear to be conserved, indicating that they could form a functional active site with an adjacent protomer (most likely TbORC1/CDC6) [58]. In contrast, the *cis* face of *T. brucei* ORC4 lacks a number of conserved and functionally important residues, suggesting that it cannot contribute to a functional active site. Perhaps this reflects the absence of further ORC subunits in *T. brucei* such that TbORC4 acts as a “book-end” in the overall ORC assembly. If correct, the ATPase activity in *T. brucei* ORC is concentrated in TbORC1/CDC6 (this protein shows conservation of all motifs), suggesting conformational changes here act in TbMCM recruitment.

The above suggestions are consistent with findings in humans, where Walker A (ATP binding) mutations in Orc4 (and Orc5) impair ORC assembly *in vitro* [17]. However, ATPase functionality for Orc4 may not be universal, since *S. cerevisiae* Orc4 also has a degenerate Walker A motif (Fig. 4); here Orc1 and Orc5 may be the focus for ATP binding [12], [66]. Moreover, *D. melanogaster* ORC forms and binds DNA when Orc4 or Orc5 are mutated in the Walker A box [67]. It may then be that *T. brucei* does indeed possess a multiprotein ORC, of which Tb7980, Tb3120 and TbORC1b may constitute further subunits. Nevertheless, the considerable sequence divergence of these factors might indicate that the detailed architecture and functioning of ORC can be tailored to the specific needs of individual organisms. While there is no clear orthology between any *T. brucei* nuclear replication factor thus far identified and Orc2, Orc3, Orc5 or Orc6, the analysis we present here on the phyletic distribution of protistan ORC factors suggests that these ORC components are less constrained in evolution than Orc1 and/or Cdc6 and Orc4. Indeed, Orc3 and Orc2 have already been considered to show greater divergence than Orc1, Orc4 and Orc5 within the more limited range of eukaryotes considered until now [7]. The lack of clear orthology of Tb7980, Tb3120 and TbORC1b might then simply reflect the considerable evolutionary distance of *T. brucei* from most well-studied eukaryotes. However, we do not exclude the interesting possibility that these factors provide replication-related functions specific to this eukaryotic lineage.

The severity and nature of the phenotypes observed following RNAi of TbORC4, Tb7980 and Tb3120 closely mirror those of TbORC1/CDC6 in PCF and BSF *T. brucei*, suggesting functional overlap. Whether this is also true of TbORC1b, for which RNAi has not been reported, we cannot say. As we have stressed, formally we have not demonstrated that each gene product acts in nuclear DNA replication, though we suspect it is likely that they do, given the striking phenocopying we report. Nonetheless, the nature and timing of the RNAi phenotypes are distinct between the two life cycle stages. In PCF cells, little growth impairment is seen until around 72–96 hrs (∼6–10 population doublings) post-RNAi induction, at which time anucleate cells slowly accumulate [43](M.Klingbeil, pers.com.). The greater impact that RNAi of TbORC1/CDC6 or its interacting factors has in BSF *T. brucei* is especially striking because at most we could detect (by quantitative RT-PCR) loss of around 40% of *TbORC1/CDC6* mRNA post RNAi (12 hours), compared with up to 90% loss in PCF cells. These observations are consistent with suggestions, from both RNAi and drug treatment studies, that a checkpoint monitoring progression from mitosis to cytokinesis is absent in PCF *T. brucei* but present in BSF cells [68], [69]. Though no work to date has examined the link between nuclear DNA replication and cell cycle checkpoints in *T. brucei*, perturbation of ORC function activates DNA damage and spindle checkpoints in yeast [70], [71]. Despite this, we cannot yet exclude the possibility that the RNAi phenotypes we see reflect non-replication functions [72] for TbORC1/CDC6 and for the ORC1/CDC6-interacting factors, and perhaps the greater severity of RNAi in BSF cells is because these non-replication roles assume greater prominence in mammal life cycle stages.

An intriguing feature of ORC in *T. brucei*, as well as in *G. lamblia* [54] and *N. gruberi* [36], is the putative absence of distinct Orc1 and Cdc6 factors. Orc1 and Cdc6 are very similar in sequence [7] and so it is highly unlikely that database searches would have found one gene but not the other, unless one had diverged significantly in sequence. As it has been argued, the fusion of Orc1 and Cdc6 functions in a single protein may be representative of an ancestral molecule, as is found in archaea [43]. However, it must now be considered that *T. brucei* ORC1/CDC6 does not function in isolation, but as part of an ORC containing at least one other factor. The functional consequences of this unusual arrangement are unclear. In other eukaryotes, Cdc6 becomes associated with ORC in a cell cycle-specific manner, inducing ATP-dependent conformational changes that cause recruitment of the MCM helicase, via Cdt1 [12], [16], [24], [28], [73]. Expression of *T. brucei* and *T. cruzi* ORC1/CDC6 is not cell cycle-dependent; instead the protein associates with chromatin in all cell cycle stages [43]. This appears to rule out the possibility that TbORC1/CDC6 is functionally related to Cdc6 and not Orc1 [21]. However, if Cdc6 is constitutively fused to ORC, this raises questions regarding how and when the MCM replicative helicase is recruited, with implications for the regulation of origin firing in these parasites. This is a problem shared with archaea, and we considered the possibility that *T. brucei* ORC might interact directly with MCM. While our interaction data are compatible with *T. brucei* MCM(2–7) possessing a conventional eukaryotic heterohexameric MCM structure with a conserved subunit interaction network, we were unable to detect interactions between TbORC1/CDC6 and TbMCM, using a number of approaches. More specifically, IP of HA-tagged TbMCM3, TbMCM6 or TbMCM7 did not reveal coIP of myc-tagged TbORC1/CDC6 from whole cells extracts. Conversely, IP of myc-tagged TbORC1/CDC6 did not coIP HA-tagged TbMCM2 or TbMCM7. Finally, IP and mass-spectrometry analyses of HA-tagged MCM subunits or Myc-tagged TbORC1/CDC6 did not reveal evidence for interaction. These data do not agree with experiments from Dang and Li [45], who observed interaction between epitope tagged TBMCM3 and both TbORC1/CDC6 and TbORC1b. Why these analyses should reach different conclusions, especially when both tested TbMCM3-TbORC1/CDC6 interaction, is unclear. It is possible that any ORC-MCM interaction is limited to the nucleus or to specific cell-cycle stages, and this may be obscured by analysing whole cell extracts, as we did. Alternatively, any such interaction may be relatively weak or transient and not maintained by the IP conditions we employed. However, unlike in archaea, where direct Orc1/Cdc6 interaction has been observed in a number of species, interaction between ORC and MCM in eukaryotes has not previously been described, except where Cdt1 was artificially tethered to Orc1–5 [28]. It therefore remains possible that a Cdt1-like mediator is present in *T. brucei* to recruit the conventionally eukaryotic MCM helicase. Such a mediator remains to be identified, and it is unclear what component, if any, of the ORC that we describe here it might act upon. Indeed, the limited homology between the putative *T. brucei* ORC components described and the canonical six-component eukaryotic ORC suggests that further characterisation is needed of ORC in this parasite and in protists.

## Materials and Methods

### *Trypanosoma brucei* strains, growth and transformation

*T. brucei* BSF cells were all of strain Lister 427 and were used and grown at 37°C in HMI-9 medium [74]. PCF cells were of strain Lister 427 or TREU927 and were grown in SDM-79 medium [75]. Cell concentration was determined with a Neubauer haemocytometer (Weber Scientific). For transformation, PCF cells at a density of 5–10×10^6^ cells.ml^−1^ were centrifuged at 600× g. for 10 min at RT and the supernatant removed and preserved for use as “conditioned medium”. ∼2.5×10^7^ cells were resuspended in 0.5 ml of ice-cold Zimmerman medium (132 mM NaCl, 8 mM KCl, 8 mM Na_2_HPO_4_, 1.5 mM KH_2_PO_4_, 0.5 mM MgAc_2_, and 0.06 mM CaAc_2_, pH 7.5) and 10 µg of linearised DNA, in a maximum volume of 10 ul of sterile double-distilled water, was added to the cells, which were subjected to two rounds of electroporation with a Bio-Rad Gene Pulser II (1.5 kV and 25 µF capacitance). The cells were then transferred into 10 ml of pre-warmed SDM79 medium and incubated at 27°C overnight. To select for antibiotic-resistant transformants, conditioned medium was prepared by adding 10% (v/v) FCS, 15% (v/v) sterile filtered medium (see above), and 75% (v/v) SDM79 medium supplemented with appropriate antibiotics. 100 ul and 1 ml of the population of electroporated and recovered parasites was added separately to 20 ml of conditioned medium and distributed across a 96 well plate (175 µl in each well). Outgrowth of antibiotic-resistant transformant clones was monitored until 10–14 days later. Transformation of BSF cells used an AMAXA Nucleofactor kit, optimised for human T-cells according to the manufacturer's protocol (Cat# VPA-1002, Amaxa Biosystems). ∼2.5×10^7^ cells, from a culture at 1–2×10^6^ cells.ml^−1^, were resuspended in 0.5 ml ice-cold Zimmerman medium supplemented with glucose (1% w/v) and transformed with 10 µg of DNA. After nucleofection, the cells were serially diluted 1∶10, 1∶100 and 1∶1000 in 30 ml HMI-9 without antibiotic, distributed in 24 well plates (1.0 ml per well) and incubated 6–12 hrs for recovery. After recovery, 1 ml of HMI-9 medium containing antibiotic was added to each well and outgrowth of transformants monitored for up to 7–10 days. DNA to be transformed was linearised using appropriate restriction enzymes in a final reaction volume of 300 µl containing ∼100 µg of DNA; the reaction mixture was incubated overnight, ethanol precipitated, resuspended in sterile double-distilled H_2_0 and the DNA concentration measured using a Nanodrop spectrophotometer (ThermoScientific).

### Epitope tagging

All proteins in this study were C-terminally epitope tagged, using constructs derived from the plasmid pNAT^12MYC^ [76]. To Myc tag TbORC1/CDC6, a C-terminal coding fragment of the ORF, excluding the stop codon, was PCR-amplified with the primers CTOL43 and CTOL44 (all primer sequences are available on request) and cloned into the above vector. The resulting construct was digested with *Xho*I and transformed in TREU927 PCF cells, which were selected with 10 µg.ml^−1^ blasticidin. TbMCM subunits were C-terminally HA tagged by modifying the pNAT^12MYC^ vector: the *blasticidin resistance* gene was replaced by *phleomycin resistance*, and the 12Myc-encoding sequence replaced with a sequence encoding 6 repeats of the HA epitope; C-terminal fragments of each *TbMCM* subunit gene were then PCR-amplified (primers: CTOL55 and CTOL56, *MCM2*; CTOL61 and CTOL62, *MCM3*; CTOL59 and CTOL60, *MCM4*; CTOL57 and CTOL58, *MCM5*; CTOL51 and CTOL52, *MCM6*; and CTOL53 and CTOL54, *MCM7*) and cloned into the resulting HA-tagging vector, replacing the *TbORC1/CDC6* fragment. The resulting constructs were digested with *Cla*I, *Hpa*I, *Xho*I, *Pvu*II, *Pvu*II and *Age*I, respectively, prior to transformation in TREU927 PCF *T. brucei*, and transformants selected with 10 µg.ml^−1^ zeocin.

### RNAi analysis

*T. brucei* PCF strain 427 pLew29-pLew13, and BSF form strain pLew90-pLEW13, developed by Wirtz *et al* [52]were used, constitutively co-expressing T7 RNA polymerase and Tet repressor. Gene fragments were amplified by PCR and cloned in to the vector pZJM [77], where they are flanked by opposing T7 promoters and Tet operator sequences. RNAi fragments were generated with the primers CTOL01 and CTOL02 (*TbORC1/CDC6*), CTOL63 and CTOL64 (*TbORC4, 13380*) or CTOL67 and CTOL68 (Tb7980). Prior to transformation, the constructs were digested with *Not*I, allowing integration into the rDNA arrays. Transformant clones were selected with 10 µg.ml^−1^ zeocin (PCF) and 2.5 µg.ml-1 phleomycin (BSF). To quantify levels of mRNA, primers CTOL7 and CTOL8 (TbORC1/CDC6), ORC4qPCRF4 and ORC4qPCRR4 (TbORC4, 13380), ORC2qPCRF and ORC2qPCRR (Tb7980) and 3120qPCRF and 3120qPCRR (Tb3120) were used; *GPI8* primers (CTOL27 and CTOL28) were used as a control. SYBR® Green PCR Master Mix (Applied Biosystems) was used for PCR in 96 well plates. A master mix for 30 reactions was made in which each reaction had 12.5 µl of SYBR mix, 1.0 µl of each primer (300 nM stock), 9.5 µl of dH_2_O, and 1.0 µl cDNA. Reactions were run on an ABI Prism 7000 thermocycler and mRNA levels quantified from amplification according to the manufacturer's instructions; conditions for all reactions were 50°C for 2 min, 95°C for 10 min, followed by 40 cycles of 95°C for 15 sec and 60°C for 1 min. For DAPI staining of *T. brucei* DNA, 5×10^5^ cells were centrifuged at 600× g, washed twice in PBS and resuspended in 100 µl of PBS. 50 µl was spread on a glass microscope slide, air-dried, and fixed in methanol at −20°C. The slides were then removed from the methanol, which was allowed to evaporate at RT, rehydrated in PBS, and Vectashield with DAPI (VectorLabs) was added. Slides were sealed with nail varnish and examined under UV light on a Zeiss Axioplan microscope. Images were captured using a Hamamatsu ORCA-ER digital camera, and Openlab version 3.0.3 software was used for processing images. For FACS analysis, 10^6^ cells were pelleted by centrifugation at 600× g, washed once in PBS, and resuspended in 70% methanol, 30% PBS. The cells were then incubated at 4°C overnight for fixation, washed in 10 ml ice-cold PBS, resuspended in 1 ml of PBS containing 10 µg.ml^−1^ propidium iodide (SIGMA) and 10 µg.ml^−1^ RNase A (SIGMA), and incubated at 37°C for 45 min. FACS was performed with a Becton Dickinson FACSCalibur using detector FL2-A and an AmpGain value of 1.75. To measure BrdU incorporation, 10^8^ PCF *T. brucei* cells were labelled with 50 µM BrdU and 50 µM 2′-deoxycytidine in SDM-79 and incubated at 27°C for 60 mins. After incubation, the cells were harvested by centrifugation at 600× g for 10 minutes, and total DNA was extracted using a Qiagen DNeasy Kit according to manufacturer's instructions. DNA samples were then incubated for 1 hr at 37°C with 33 µg.ml^−1^ RNase A (Sigma R4642). The amount of purified DNA was determined using a NanoDrop (Thermo Scientific) and 2 µg of total DNA was incubated with 10 volumes of 0.4 N NaOH solution for 30 min at room temperature (RT) and kept on ice. The DNA solution was then neutralised with an equal volume of 1 M Tris·HCl (pH 6.8) and dot-blotted (50 ng in 5 µl) onto a nitrocellulose membrane (Amersham) and allowed to air dry. The DNA was fixed twice on the membrane using an ultraviolet cross-linker Stratalinker (Stratagene) and incubated with mouse anti-BrdU monoclonal antibody (1∶2,000 dilution, B2531, Sigma) in buffer containing TBST (20 mM Tris·HCl, pH 7.6, 136 mM NaCl, and 0.05% Tween 20) and 1% non-fat milk for 1 hr at RT. The membrane was then washed with TBST three times for 10 mins each at RT, and incubated with horseradish peroxidase-conjugated anti-mouse IgG antibody (1∶5,000 dilution) for 1 h at RT. Next the membrane was washed three times again with TBS-T for 20 min at RT and BrdU signal detected either using an enhanced chemiluminescence detection system, using the QuantityOne software (BioRad), or exposure to X-Ray film.

### Immunoprecipitation

50 µl of M-280 IgG-coated magnetic Dynalbeads (Invitrogen) was pre-blocked by washing, twice, with 1 ml of block solution (0.05% BSA in PBS), each time collecting the beads using a magnetic rack and discarding the supernatant, before finally being resuspended in 250 µl block solution. 5 µg of antibody specific to the desired epitope tag was then added to the beads/block solution and incubated, rotating, at 4°C overnight. The beads were then washed 3 times in 1 ml block solution and resuspended in 100 µl block solution. Whole cell *T. brucei* extract was prepared by centrifugation of 10^8^ cells at 2000× *g* for 10 minutes. After washing twice in ice-cold PBS, the cells were resuspended in 2 ml WCE buffer (50 mM HEPES pH 7.55, 100 mM NaCl, 1.0 mM EDTA, 1.0 mM EGTA, 10% glycerol, 1% Triton X100 and protease inhibitors) and lysed on ice for 1–2 hours. The cell lysate was then centrifuged at 15000× g at 4°C for 30 mins and a sample of supernatant aliquoted and stored at −20°C to serve as ‘input’. The rest of the supernatant was added to the beads (prepared above) and incubated for 2 hrs. After incubation, the samples were washed seven times with 1.0 ml ice-cold wash buffer (50 mM HEPES pH 7.55, 500 mM LiCl, 1.0 mM EDTA, 1.0 mM EGTA, 0.7% Na deoxycholate, 1% NP40 and protease inhibitors), and then finally washed with 1 ml TE wash buffer (10 mM Tris.HCl pH 8.0, 50 mM NaCl and 1 mM EDTA). After the TE wash step, the samples were centrifuged at 1000× g for 3 mins at 4°C, residual TE wash buffer carefully removed, and incubated in 220 µl of Elution buffer (50 mM Tris.HCl pH 8.0, 10 mM EDTA, 1% SDS) at 65°C for 30 mins, with intermittent vortexing every 2–5 mins. The beads were then centrifuged for 1 min at 16,100× g at room temp. and 200 µl of the supernatant removed, which served as the ‘eluate’. For coIP analysis, 20 µl of input and eluate samples were separated by SDS-PAGE and analysed by western blotting (using anti-Myc and anti-HA monoclonal antibodies; Millipore). For protein identification, the eluate samples were also separated by SDS-PAGE and visualized by staining with colloidal coommassie; protein bands were excised and analysed by Liquid Chromatography-Electrospray Tandem Mass Spectrometry at the University of Glasgow Sir Henry Wellcome Functional Genomics facility, or at the University of Dundee proteomics facility.

### Yeast two-hybrid

Yeast two-hybrid interaction assays were carried out using the Matchmaker Gold yeast two-hybrid system (Clontech). The MCM ORFs were PCR-amplified (primers available on request) and cloned into pGADT7 and pGBKT7 vectors, which were then transformed into yeast strains Y2H Gold and Y187, respectively. Transformants were selected on SD/-Trp/-Leu media. To test interactions between MCM proteins, at least two clones of cell lines containing both vectors were plated onto SD/-Trp/-Leu/-His with and without 125 ng/ul Aureobasidin A or 2.5 mM 3′ aminotriazole. Interactions were classified as being ‘strong’ if growth occurred on both media, ‘weak’ if growth occurred only on media without Aureobasidin A or 3′ aminotriazole.

## Supporting Information

Figure S1 **Yeast 2-hybrid analysis of interactions between** ***T. brucei*** **MCM subunits.** **A.** Growth of yeast clones co-expressing individual MCM subunits (numbered 2–7, indicating MCM2–7) as fusions with the Gal DNA binding domain (pGBK-MCM) and with the Gal activation domain (pGAD-MCM) is shown (2 clones for each pair); as a contro,l the MCM-DNA binding fusions are shown co-expressed with the Gal activator domain unfused to any protein (pGAD-Empty). Growth on minimal medium lacking tryptophan, leucine and histidine (-T-L-H), or supplemented with Aureobasidin A (-T-L-H+AbA), indicates weak and strong interactions, respectively; growth on medium lacking only tryptophan and lecuine (-T-L) shows that the cells that cannot grow through interaction are viable. **B.** The MCM3-Gal DNA binding domain fusion (pGBK-MCM3 co-expressed with pGAD-Empty) appeared to show some autoactivation, so this interaction analysis was repeated, and further tested by growth on mimimal medium lacking tryptophan, leucine and histidine and supplemented with 2.5 mM 3′ aminotriazole (-T-L-H+3-AT). **C.** The MCM6-Gal activator domain fusion (pGBK-MCM6) appeared to show extensive weak interactions, and this was retested by analysing growth of four independently generated yeast clones (numbered 1–4) co-expressing the protein with MCM-Gal DNA binding domain fusions.
(PDF)

Figure S2 **A phylogenetic tree of eukaryotic ORC proteins and the novel, putative** ***T. brucei*** **ORC proteins.** A neighbour-joining phylogenetic tree is shown that was generated from a ClustalX alignment of validated or putative ORC subunit polypeptides; the lengths of the arms in the tree are proportional to the size marker, where the line length indicates 10 amino acid changes per 100 amino acids. Hsa, *Homo sapiens*; Dme, *Drosophila melanogaster*; Ath, *Arabidopsis thaliana*; Sce, *Saccharomyces cerevisiae*; Ecu, *Encephalitozoon cuniculi*; Ddi, *Dictyostelium discoideum*; Pfa, *Plasmodium falciparum*; Cpa, *Cryptosporidium parvum*; Tan, *Theileria annulata*; Tth, *Tetrahymena thermophila*; Gla, *Giardia lamblia*; Tva, *Trichomonas vaginalis*; Ehi, *Entamoeba histolytica*; Tps, *Thalassiosira pseudonona*; Tbr, *Trypanosoma brucei*; Lma, *L. major*; Tcr, *T. cruzi*. Genbank accession numbers are provided in Text S1.
(PDF)

Figure S3 **RNAi of TbORC1/CDC6, TbORC4, Tb7980 and Tb3120 in procyclic form** ***T. brucei*** **cells.** Growth curves are shown for procyclic form *T. brucei* cells in the absence or presence of tetracycline (tet−, shown as solid line, and tet+, dashed line, respectively), which induces RNAi, targeting either *TbORC1/CDC6*, *TbORC4 (Tb13380)*, *Tb7980* or *Tb3120* mRNA. For each factor, cell density over time was examined and cell counts are shown from 3 days post-RNAi induction.
(PDF)

Figure S4 Representative images of procyclic form *T. brucei* cells after RNAi induction against *TbORC1/CDC6*, *TbORC4 (13380)*, *Tb7980* or *Tb3120* are shown 6 days post RNAi-induction; all images are shown as an overlay of DAPI-stained and phase images, and arrows highlight ‘zoid’ cells that lack nuclear DNA but retain kDNA.
(PDF)

Figure S5 **Comparison of RNAi phenotypes at two different levels of ORC1/CDC6 mRNA knockdown in procyclic form** ***T. brucei*** **.** Quantitative reverse-transcriptase PCR (qRT-PCR) to determine TbORC1/CDC6 mRNA levels after RNAi is shown (left) for cells in which RNAi leads to ∼90% loss of mRNA (top) and 75% loss (bottom), 96 hours post-RNAi induction. The abundance of TbORC1/CDC6 cDNA from RNAi-induced cells (Tet+, black bar) is shown to relative to control cells without TbORC1/CDC6 RNAi (tet−, grey bar). The concentration of PCR product in the non-induced sample is normalised to 1.0; values are the means from at least three experimental repetitions and vertical lines denote standard deviation. In the middle, histograms are shown of propidium iodide-stained (PI) DNA from cells after FACS sorting, sampled pre- and post - induction of RNAi against TbORC1/CDC6 (−Tet and+Tet, respectively); the histograms refer to the 90% and 75% RNAi cells to the left. Peaks corresponding with cells containing 2C and 4C DNA content are indicated, as is the peak position for cells with 8C content (C represents haploid DNA content). The rightmost diagram shows dot-blots of *T. brucei* DNA probed with anti-BrdU antibody. DNA is shown from the cells incubated with BrdU after RNAi was induced by tetracycline (+Tet) for 96 or 144 hrs, targeted against either TbORC1/CDC6 or Tb927.6.5070 (as a control); in all cases RNAi-induced cells are compared with control cells in which RNAi was not induced (−Tet). As before, the TbORC1/CDC6 dot blots refer to the 90% and 75% RNAi cells shown the far left; RNAi against Tb927.6.5070 was quantified by qRT-PCR and shown to reduce mRNA levels by ∼90% (data not shown).
(PDF)

Figure S6 Representative images of aberrant bloodstream form *T. brucei* cells after RNAi induction against *TbORC1/CDC6*, *TbORC4 (13380)* or *Tb7980* are shown at the time points indicated; DAPI stain (DAPI), phase contrast (PHASE), and an overlay of the DAPI and phase images (MERGE) are indicated.
(PDF)

Text S1 **Accession or genome identification numbers for the putative Origin Recognition Complex subunits used in this study.**
(PDF)
