# Supplementary figures and images for: Identification of ORC1/CDC6-Interacting Factors in Trypanosoma brucei Reveals Critical Features of Origin Recognition Complex Architecture
Source: PLoS One. 2012 Mar 8;7(3):e32674. doi: 10.1371/journal.pone.0032674 (PMC3297607; doi:10.1371/journal.pone.0032674)

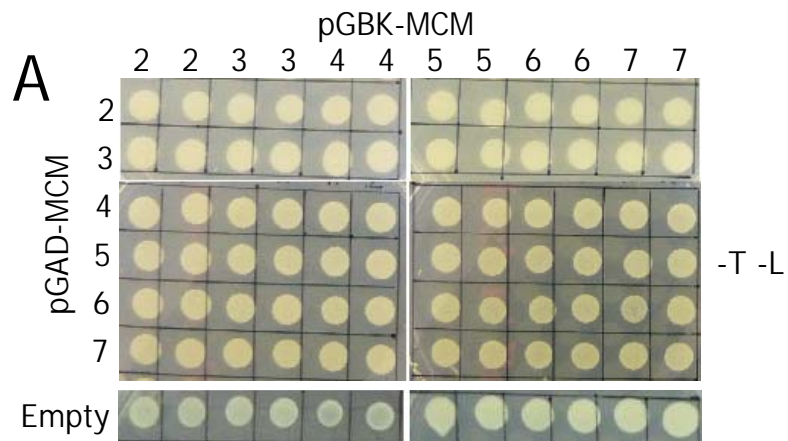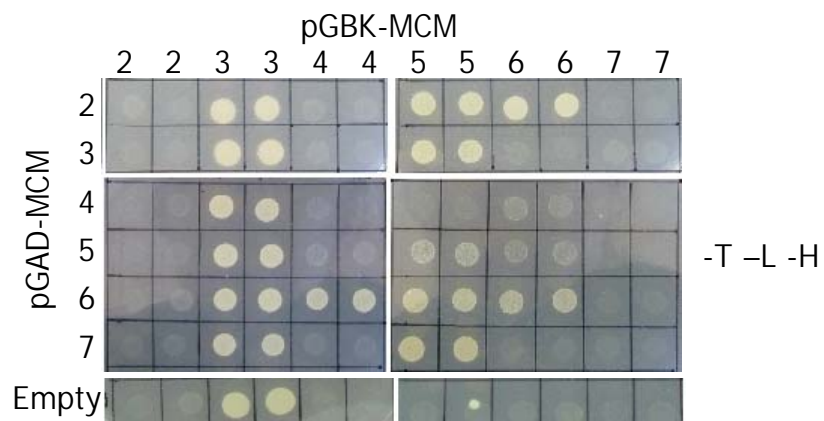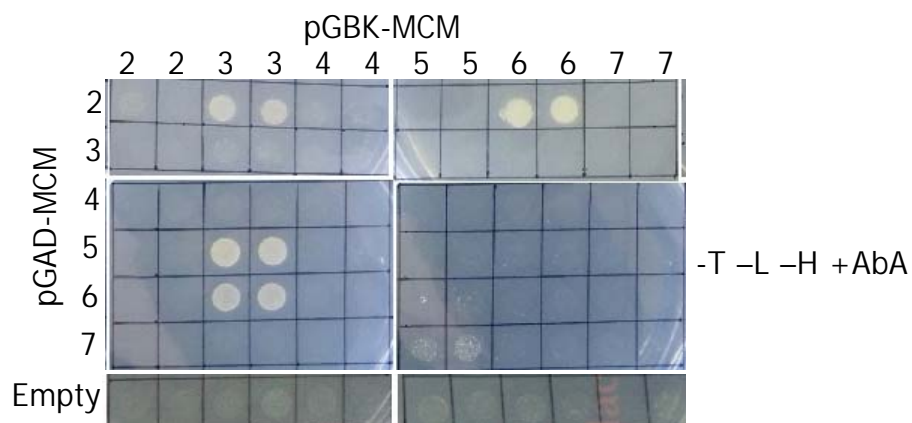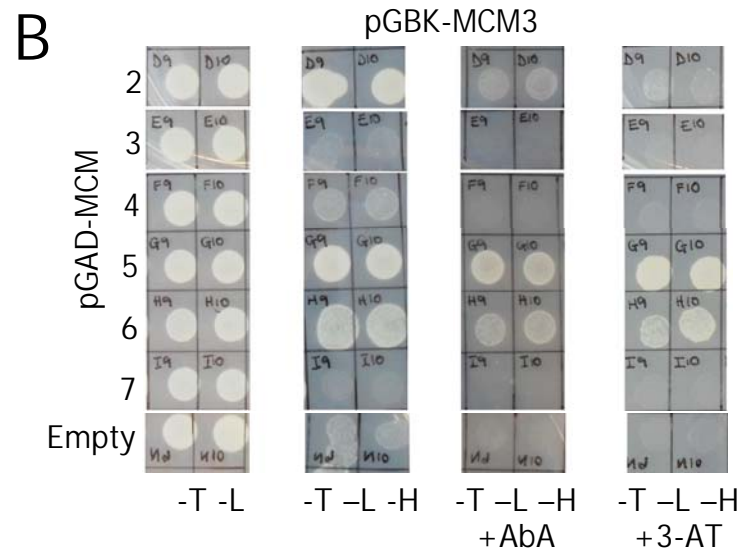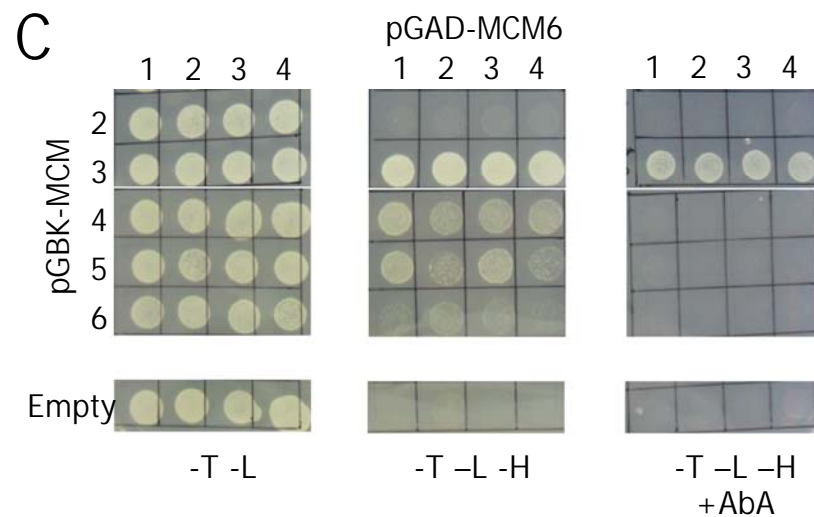

Supplement: Figure S1 — Yeast 2-hybrid analysis of interactions between T. brucei MCM subunits. A. Growth of yeast clones co-expressing individual MCM subunits (numbered 2–7, indicating MCM2–7) as fusions with the Gal DNA binding domain (pGBK-MCM) and with the Gal activation domain (pGAD-MCM) is shown (2 clones for each pair); as a contro,l the MCM-DNA binding fusions are shown co-expressed with the Gal activator domain unfused to any protein (pGAD-Empty). Growth on minimal medium lacking tryptophan, leucine and histidine (-T-L-H), or supplemented with Aureobasidin A (-T-L-H+AbA), indicates weak and strong interactions, respectively; growth on medium lacking only tryptophan and lecuine (-T-L) shows that the cells that cannot grow through interaction are viable. B. The MCM3-Gal DNA binding domain fusion (pGBK-MCM3 co-expressed with pGAD-Empty) appeared to show some autoactivation, so this interaction analysis was repeated, and further tested by growth on mimimal medium lacking tryptophan, leucine and histidine and supplemented with 2.5 mM 3′ aminotriazole (-T-L-H+3-AT). C. The MCM6-Gal activator domain fusion (pGBK-MCM6) appeared to show extensive weak interactions, and this was retested by analysing growth of four independently generated yeast clones (numbered 1–4) co-expressing the protein with MCM-Gal DNA binding domain fusions. (PDF) [file pone.0032674.s001.pdf]

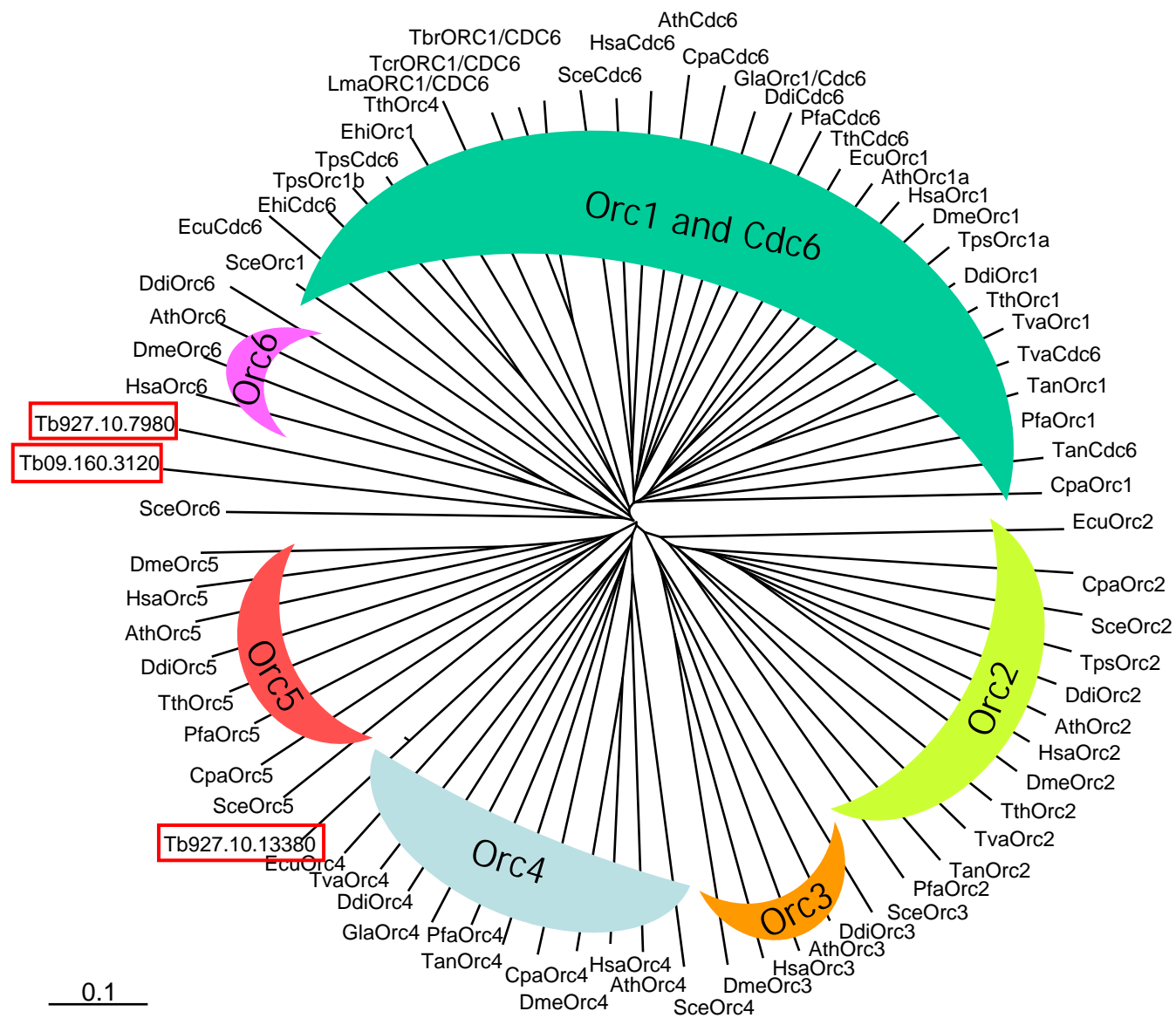

Suppl. Fig. 2

Supplement: Figure S2 — A phylogenetic tree of eukaryotic ORC proteins and the novel, putative T. brucei ORC proteins. A neighbour-joining phylogenetic tree is shown that was generated from a ClustalX alignment of validated or putative ORC subunit polypeptides; the lengths of the arms in the tree are proportional to the size marker, where the line length indicates 10 amino acid changes per 100 amino acids. Hsa, Homo sapiens; Dme, Drosophila melanogaster; Ath, Arabidopsis thaliana; Sce, Saccharomyces cerevisiae; Ecu, Encephalitozoon cuniculi; Ddi, Dictyostelium discoideum; Pfa, Plasmodium falciparum; Cpa, Cryptosporidium parvum; Tan, Theileria annulata; Tth, Tetrahymena thermophila; Gla, Giardia lamblia; Tva, Trichomonas vaginalis; Ehi, Entamoeba histolytica; Tps, Thalassiosira pseudonona; Tbr, Trypanosoma brucei; Lma, L. major; Tcr, T. cruzi. Genbank accession numbers are provided in Text S1. (PDF) [file pone.0032674.s002.pdf]

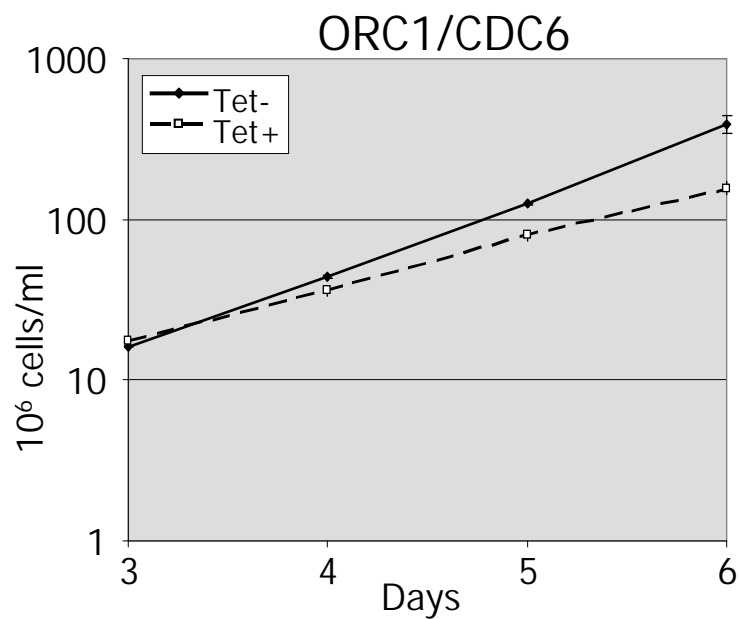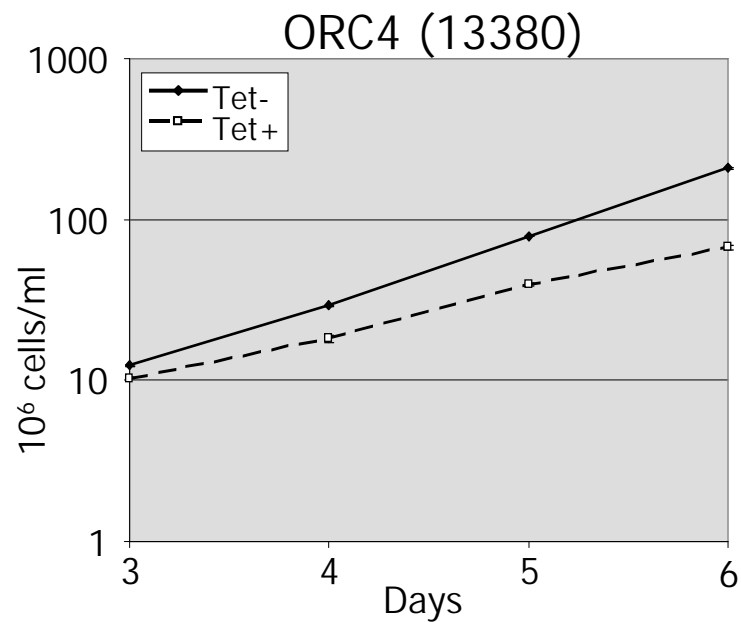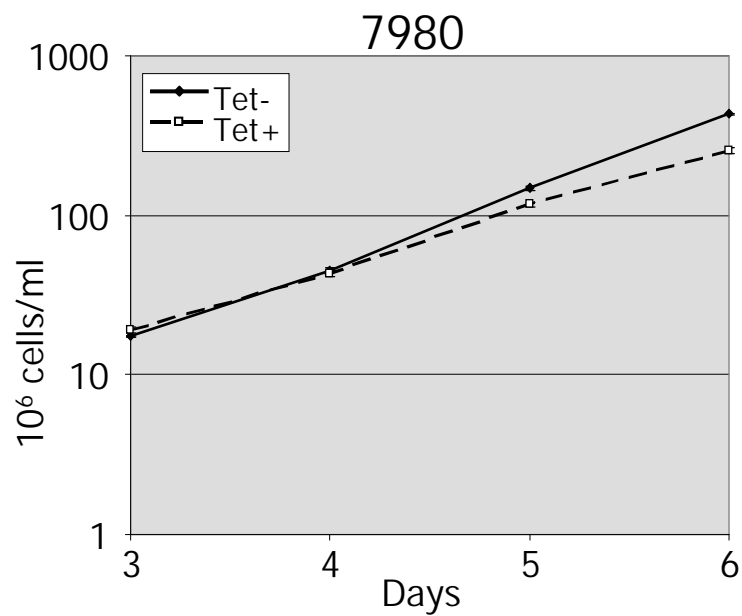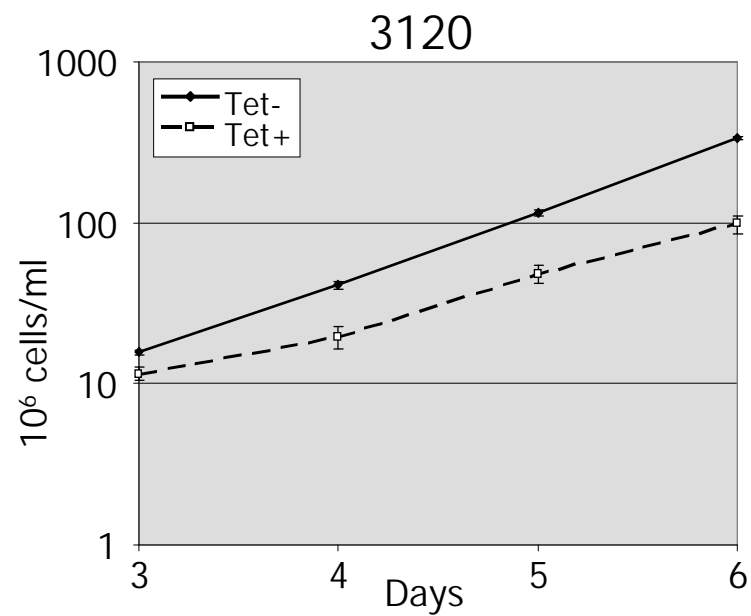

Suppl. Fig. 3

Supplement: Figure S3 — RNAi of TbORC1/CDC6, TbORC4, Tb7980 and Tb3120 in procyclic form T. brucei cells. Growth curves are shown for procyclic form T. brucei cells in the absence or presence of tetracycline (tet−, shown as solid line, and tet+, dashed line, respectively), which induces RNAi, targeting either TbORC1/CDC6, TbORC4 (Tb13380), Tb7980 or Tb3120 mRNA. For each factor, cell density over time was examined and cell counts are shown from 3 days post-RNAi induction. (PDF) [file pone.0032674.s003.pdf]

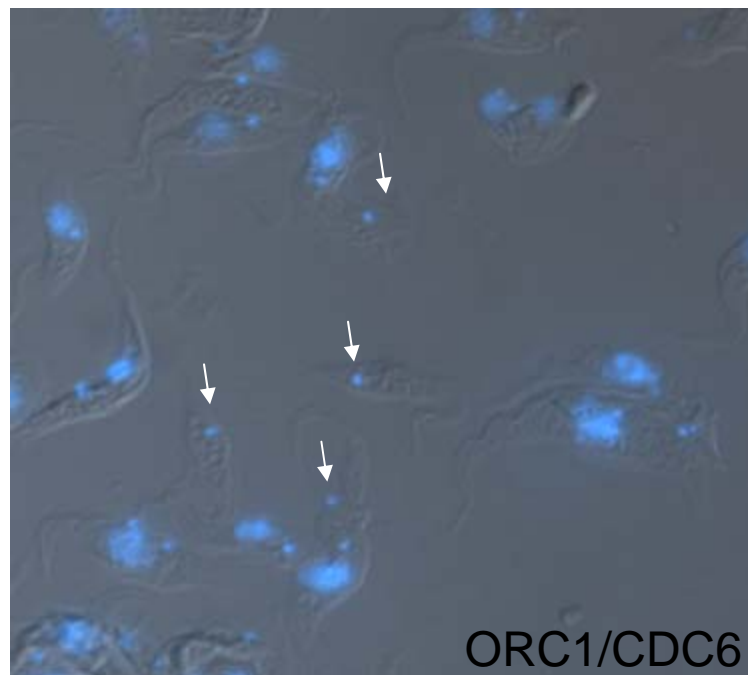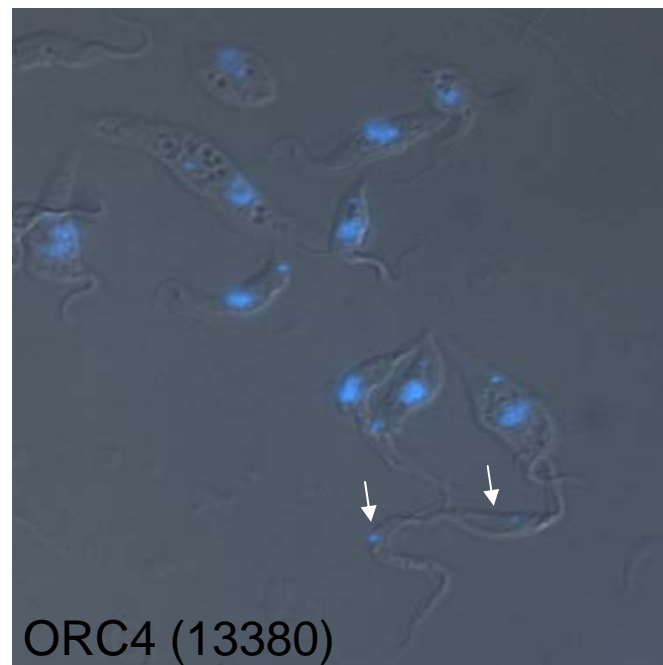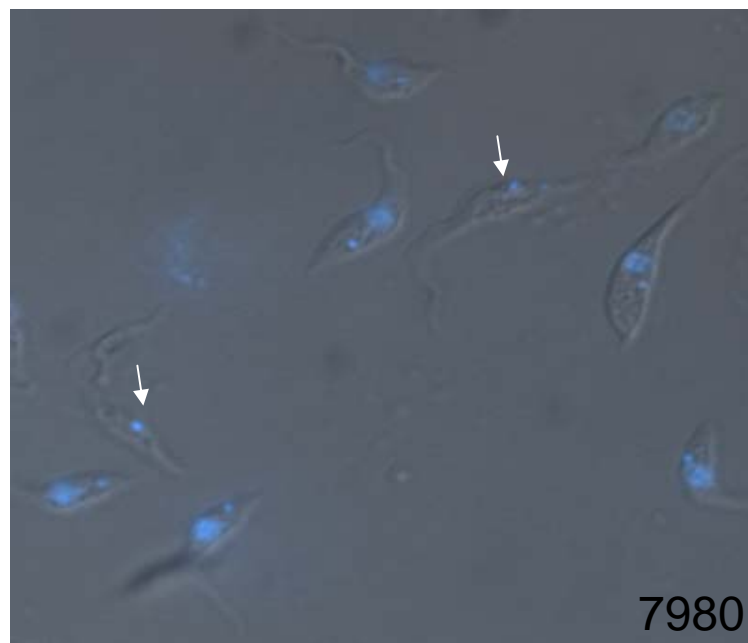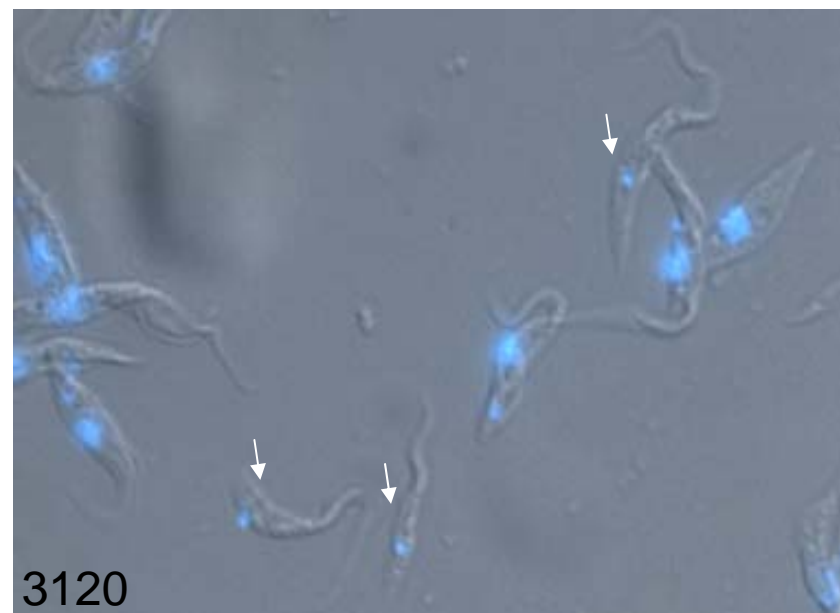

Suppl. Fig. 4

Supplement: Figure S4 — Representative images of procyclic form T. brucei cells after RNAi induction against TbORC1/CDC6, TbORC4 (13380), Tb7980 or Tb3120 are shown 6 days post RNAi-induction; all images are shown as an overlay of DAPI-stained and phase images, and arrows highlight ‘zoid’ cells that lack nuclear DNA but retain kDNA. (PDF) [file pone.0032674.s004.pdf]

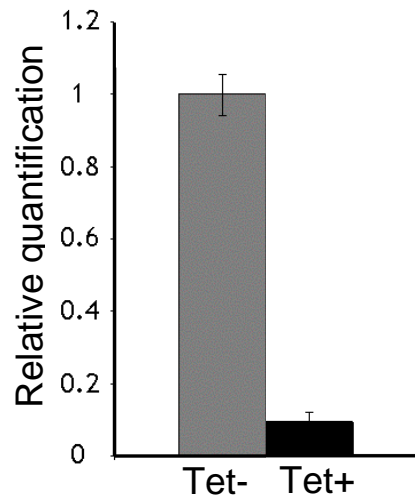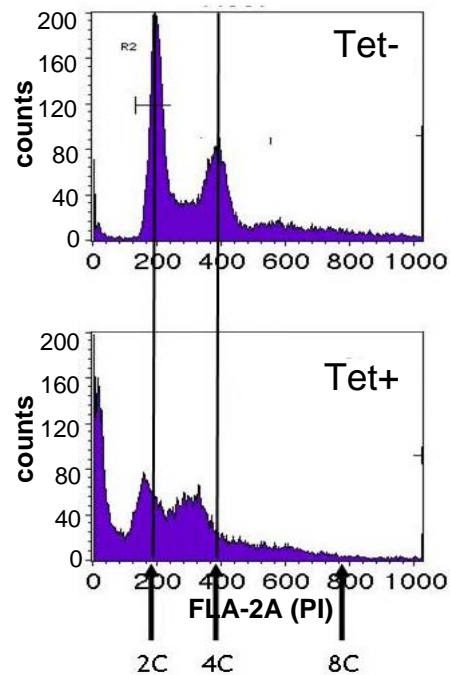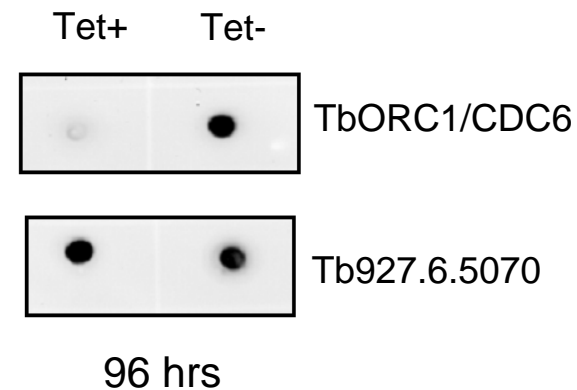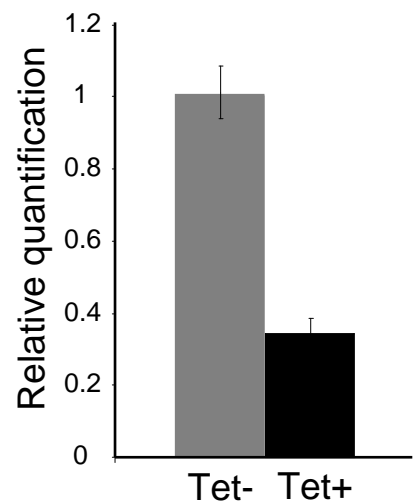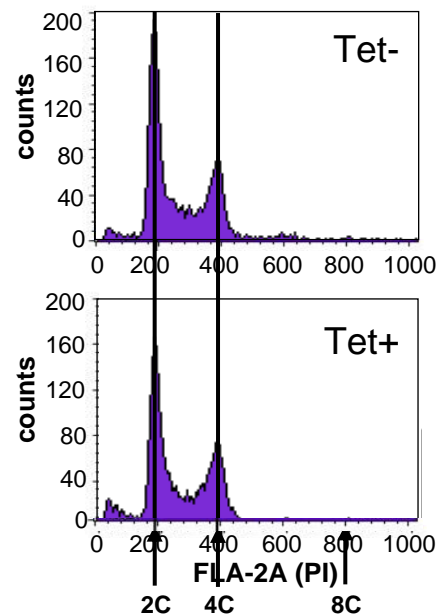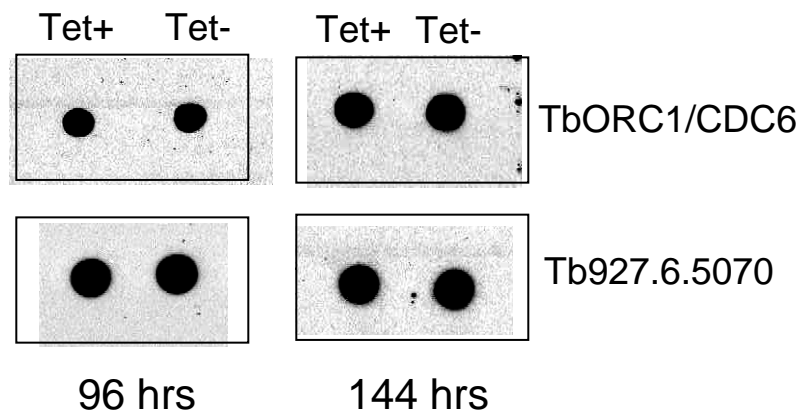

Suppl. Fig. 5

Supplement: Figure S5 — Comparison of RNAi phenotypes at two different levels of ORC1/CDC6 mRNA knockdown in procyclic form T. brucei . Quantitative reverse-transcriptase PCR (qRT-PCR) to determine TbORC1/CDC6 mRNA levels after RNAi is shown (left) for cells in which RNAi leads to ∼90% loss of mRNA (top) and 75% loss (bottom), 96 hours post-RNAi induction. The abundance of TbORC1/CDC6 cDNA from RNAi-induced cells (Tet+, black bar) is shown to relative to control cells without TbORC1/CDC6 RNAi (tet−, grey bar). The concentration of PCR product in the non-induced sample is normalised to 1.0; values are the means from at least three experimental repetitions and vertical lines denote standard deviation. In the middle, histograms are shown of propidium iodide-stained (PI) DNA from cells after FACS sorting, sampled pre- and post - induction of RNAi against TbORC1/CDC6 (−Tet and+Tet, respectively); the histograms refer to the 90% and 75% RNAi cells to the left. Peaks corresponding with cells containing 2C and 4C DNA content are indicated, as is the peak position for cells with 8C content (C represents haploid DNA content). The rightmost diagram shows dot-blots of T. brucei DNA probed with anti-BrdU antibody. DNA is shown from the cells incubated with BrdU after RNAi was induced by tetracycline (+Tet) for 96 or 144 hrs, targeted against either TbORC1/CDC6 or Tb927.6.5070 (as a control); in all cases RNAi-induced cells are compared with control cells in which RNAi was not induced (−Tet). As before, the TbORC1/CDC6 dot blots refer to the 90% and 75% RNAi cells shown the far left; RNAi against Tb927.6.5070 was quantified by qRT-PCR and shown to reduce mRNA levels by ∼90% (data not shown). (PDF) [file pone.0032674.s005.pdf]

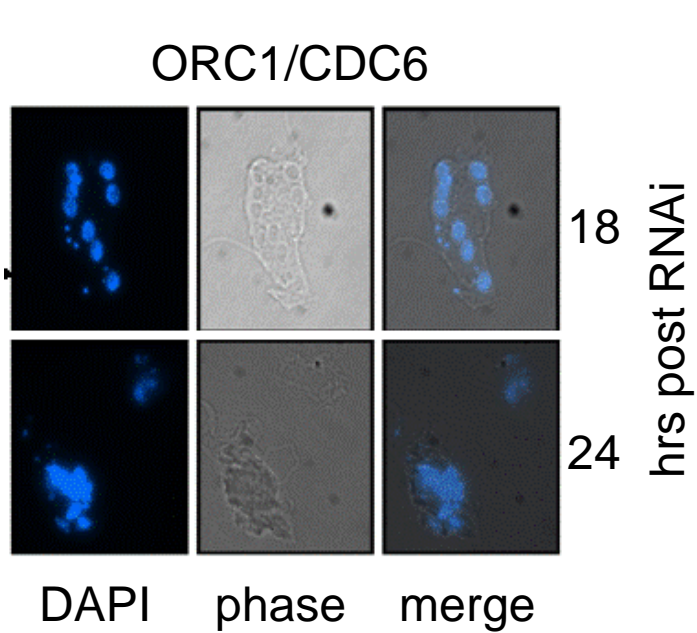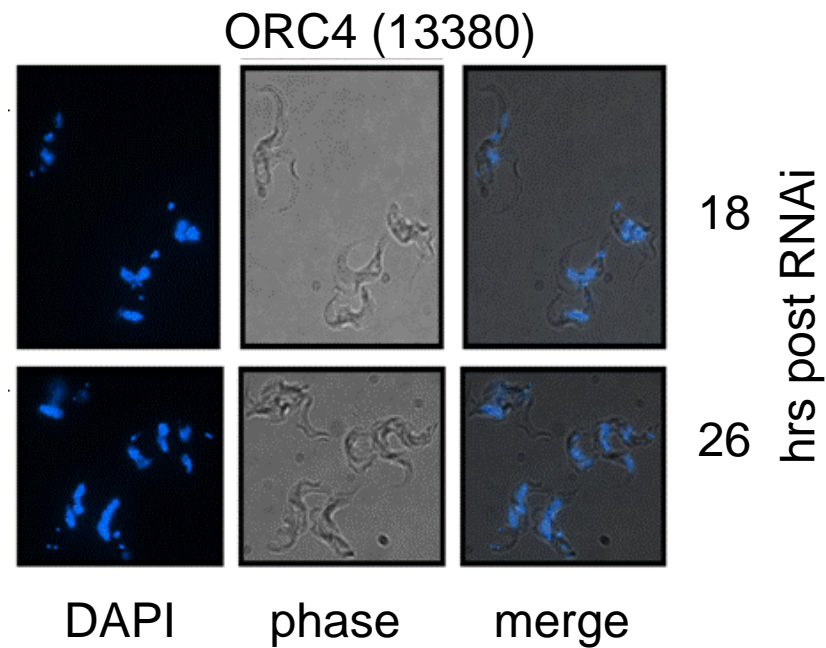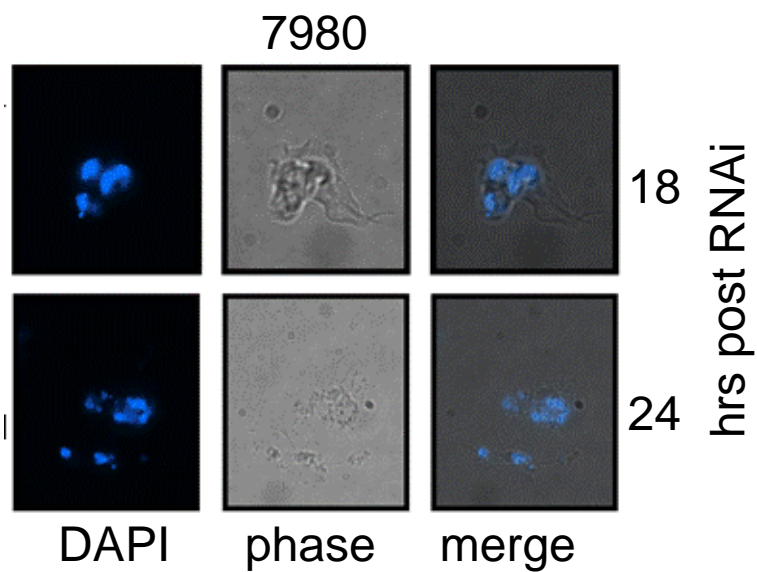

Supplement: Figure S6 — Representative images of aberrant bloodstream form T. brucei cells after RNAi induction against TbORC1/CDC6, TbORC4 (13380) or Tb7980 are shown at the time points indicated; DAPI stain (DAPI), phase contrast (PHASE), and an overlay of the DAPI and phase images (MERGE) are indicated. (PDF) [file pone.0032674.s006.pdf]
