# Supplementary material for: Identification of ORC1/CDC6-Interacting Factors in Trypanosoma brucei Reveals Critical Features of Origin Recognition Complex Architecture
Source: PLoS One. 2012 Mar 8;7(3):e32674. doi: 10.1371/journal.pone.0032674 (PMC3297607; doi:10.1371/journal.pone.0032674)
Supplement: Text S1 — Accession or genome identification numbers for the putative Origin Recognition Complex subunits used in this study. (PDF) [file pone.0032674.s007.pdf]

Identification of ORC1/CDC6-interacting factors in *Trypanosoma brucei* reveals critical features of Origin Recognition Complex architecture

Calvin Tiengwe[1], Lucio Marcello[1], Helen Farr[2], Catarina Gadelha[2,3], Richard Burchmore[4], J. David Barry[1], Stephen D. Bell[2] and Richard McCulloch[1]*

1.University of Glasgow, College of Medical Veterinary and Life Sciences, Institute of Infection, Immunity and Inflammation, The Wellcome Trust Centre for Molecular Parasitology, Sir Graeme Davies Building, 120 University Place, Glasgow, G12 8TA, U.K.

2.University of Oxford, Sir William Dunn School of Pathology, South Parks Road, Oxford, OX1 3RE, U.K.

3.Present address: Department of Pathology, University of Cambridge, Tennis Court Road, Cambridge, CB2 1QP,U.K.

4.University of Glasgow, College of Medical Veterinary and Life Sciences, Institute of Infection, Immunity and Inflammation, Sir Henry Wellcome Functional Genomics Faciltiy, RB227B Level B2, Joseph Black Building, Glasgow, G12 8QQ, U.K.

Running title: Origin Recognition Complex factors in *Trypanosoma brucei*

Keywords: trypanosome; replication; origin; origin recognition complex; pre-replication complex; MCM helicase

*Address correspondence to: Richard McCulloch. Tel: 0(44)141-330-5946. Fax: 0(44)141-330-5422). Email: **richard.mcculloch@glasgow.ac.uk**

Accession, or genome identification, numbers for the putative Origin Recognition Complex subunits used in this study.

**1. Excavata.**	
Trichomonas vaginalis	**(http://trichdb.org/trichdb/)**
Orc1	TVAG_340580
Orc2	TVAG_185790
Orc4	TVAG_365960
Cdc6	TVAG_376490
***Giardia intestinalis* (lamblia)**	**(http://giardiadb.org/giardiadb/)**
Orc1/Cdc6	ctg02_20
Orc4	ctg02_3
Trypanosoma brucei	**(http://tritrypdb.org/tritrypdb/)**
ORC1/CDC6	Tb11.02.5110(EAN79686)
Leishmania major	**(http://tritrypdb.org/tritrypdb/)**
ORC1/CDC6	F28.0030(CAJ04600)
Trypanosoma cruzi	**(http://tritrypdb.org/tritrypdb/)**
ORC1/CDC6	Tc00.1047053511159.20(XP_806474)
Naegleria gruberi	**(http://genome.jgi-psf.org/Naegr1/Naegr1.home.html)**
Orc1/Cdc6	jgi|Naegr1|88228|LAD_fgeneshNG_pg.scaffold_35000178
Orc4	jgi|Naegr1|45184|fgeneshHS_pg.scaffold_1000274
Orc2	jgi|Naegr1|88227|LAD_fgenesh2_pg.C_scaffold_19000304
Orc6	jgi|Naegr1|88231|LAD_query
**2. Chromalveolata**	
Thalassiosira pseudonona	**(http://genome.jgi-psf.org/Thaps3/Thaps3.home.html)**
Orc1a	Thaps3|263879|thaps1_ua_kg.chr_12000030
Orc1b (Cdc6b)	fgenesh1_pg.C_chr_6000022 [Thaps3:5867]
Orc2	jgi|Thaps3|260866|thaps1_ua_kg.chr_1000126
Cdc6	jgi|Thaps3|8536|fgenesh1_pg.C_chr_10000306
Cryptosporidium parvum	**(http://cryptodb.org/cryptodb/)**
Orc1	cgd4_4320
Orc2	cgd4_1930
Orc4	cgd2_1550
Orc5	cgd4_430
Cdc6	cgd7_2310
Theileria annulata	**(http://old.genedb.org/genedb/annulata/)**
Orc1	TA04740
Orc2	TA19765
Orc4	TA12985
Cdc6	TA14555
Plasmodium falciparum	**(http://plasmodb.org/plasmo/)**
Orc1	PFL0150w
Orc2	MAL7P1.21
Orc4	PF13_0189
Orc5	PFB0720c
Cdc6	PFE0155w
Tetrahymena thermophila	**(http://ciliate.org/index.php/home/welcome)**
Orc1	154.m00093
Orc2	104.m00101
Orc4	51.m00235
Orc5	142.m00114
Cdc6	50.m00158

3. Plantae

Arabidopsis thaliana	
Orc1a	CAD13174
Orc2	Q38899
Orc3	AAT37463
Orc4	CAE01428
Orc5	NP_194720
Orc6	Q9ZVH3
Cdc6	CAC81074

4. Amoebozoa

Entamoeba histolytica	**(http://www.sanger.ac.uk/cgi-bin/blast/submitblast/e_histolytica)**
Orc1	EHI_105150
Cdc6	EHI_192770
Dictyostelium discoideum	**(http://dictybase.org/)**
Orc1	DDB0218435
Orc2	DDB0190652
Orc3	DDB0216767
Orc4	DDB0168430
Orc5	DDB0191826
Orc6	DDB0186183
Cdc6	DDB0203551

5. Opisthokonta

Encephalitozoon uniculi	**(http://microsporidiadb.org/micro/)**
Orc1	NP_597612
Orc2	NP_586228
Orc5	NP_597618
Cdc6	XP_955574
Saccharomyces cerevisiae	
Orc1	P54784
Orc2	P32833
Orc3	P54790
Orc4	P54791
Orc5	P50874
Orc6	P38826
Cdc6	NP_012341
Drosophila melanogaster	
Orc1	AAF59236.1
Orc2	AAF55006.1
Orc3	AAD39472.1
Orc4	AAF47276.1
Orc5	AAF53340.1
Orc6	AAF58890.1
Homo sapiens	
Orc1	Q13415
Orc2	Q13416
Orc3	Q9UBD5
Orc4	O43929
Orc5	O43913
Orc6	Q9Y5N6
Cdc6	NP_001245
